# ﻿New species of *Papuanatula* Lugo-Ortiz & McCafferty, 1999 from New Guinea (Ephemeroptera, Baetidae) with focus on Batanta Island

**DOI:** 10.3897/zookeys.1259.168419

**Published:** 2025-11-10

**Authors:** Thomas Kaltenbach, Tibor Kovács, Jean-Luc Gattolliat

**Affiliations:** 1 Muséum cantonal des Sciences Naturelles, Département de zoologie, Palais de Rumine, Place Riponne 6, CH-1005 Lausanne, Switzerland Muséum cantonal des Sciences Naturelles Lausanne Switzerland; 2 University of Lausanne (UNIL), Department of Ecology and Evolution, CH-1015 Lausanne, Switzerland University of Lausanne (UNIL) Lausanne Switzerland; 3 Mátra Museum of the Hungarian Natural History Museum of the Hungarian National Museum Public Collection Centre, Kossuth Lajos u. 40, H-3200 Gyöngyös, Hungary Mátra Museum of the Hungarian Natural History Museum of the Hungarian National Museum Public Collection Centre Gyöngyös Hungary

**Keywords:** COI, eggs, imago, *
Papuafiliola*, subimago, West Papua

## Abstract

Since 2010, the Mátra Museum of the Hungarian Natural History Museum has conducted a long-term research program to explore the biodiversity and collect data of selected groups of insects and partly other animals on the island of Batanta (Indonesia, West Papua). During this research project, numerous mayflies of different stages were collected as well. In this study, mayflies of the highly diverse genus *Papuanatula* (Ephemeroptera, Baetidae) collected in Batanta between 2014 and 2025 were investigated. Six new species were discovered, four belonging to the subgenus Papuanatula s. str., *P. batantaraja***sp. nov.**, *P. cukiclara***sp. nov.**, *P. cataracta***sp. nov.**, *P. batanlenos***sp. nov.**, and two to the subgenus Papuafiliola, *P. horvathrobi***sp. nov.** and *P. longabranchias***sp. nov.** Their larval stages are described and illustrated. Additionally, imagos and subimagos of two of the new species are described as well, and the eggs of one species. Furthermore, a new species of *Papuanatula* from another region of New Guinea is described. For most species, the species delimitation is also supported by mitochondrial DNA data (COI). Keys to the larvae of all species per subgenus or species group relevant for Batanta are presented. The total number of *Papuanatula* species is augmented to 33.

## ﻿Introduction

Currently, the Mátra Museum of the Hungarian Natural History Museum in collaboration with other research organizations is running a research program on the biodiversity of Batanta Island (Indonesia, New Guinea, West Papua). Covering an area of approximately 450 km^2^, Batanta is a small island largely covered by tropical lowland rainforest, with rich and undisturbed forest streams, its highest point is 1184 m (for details see Kovács et al. 2015). The research program is primarily focused on various insect groups; it started in 2010 and is planned to continue for several more years. From 2014, every year a research group (including one of us, TiKo) has been conducting several weeks collecting and observation campaigns in various parts of Batanta, with the support of locals. Since the beginning of this program, a number of articles presenting results from different research fields have been published: Odonata, with one species known before, 64 species were recorded by Kovács and Theischinger (2023), including seven species new to science, and three additional new species will be described in another article (Kovács and Theischinger in preparation); Trichoptera, with no known species, 11 articles were published with data on 163 species of which 157 were described as new species (Kovács et al. 2025). Other groups were studied as well, such as epiphyllous liverworts (Marchantiophyta), for which no data were available before, 48 taxa were found, including a new species and a new subspecies (Pócs and Kovács 2023); earthworms (Megadrili: Acanthodrilidae, Megascolecidae), for which no data were available before, five species were identified (Szederjesi 2019); Gastropoda (Cyclophoroidea, Pupinidae), Varga and Páll-Gergely (2017) described *Bellardiella kovacsi* as a new species; most recently, the distribution and behaviour of Wilson’s bird-of-paradise (*Diphyllodes respublica*) was reported by Horváth et al. (2024).

In the present study, we examine the material of the highly diverse genus *Papuanatula* Lugo-Ortiz & McCafferty, 1999 (Ephemeroptera, Baetidae) collected between 2014 and 2025 in Batanta. Presently, *Papuanatula* is divided in two subgenera, *Papuanatula* s. str. and *Papuafiliola* Kaltenbach, Kluge & Gattolliat, 2025. Further, *Papuanatula* s. str. is consisting of five species groups defined in Kaltenbach et al. (2025). We recorded six different species of Papuanatula in Batanta, four belonging to the subgenus Papuanatulas. str. and two to the subgenus Papuafiliola, all of them being new to science. They are described and illustrated mainly based on larvae, but partly also based on imagos, subimagos and eggs. Species delimitation is mostly supported by mitochondrial DNA data (COI) as well. Additionally, we describe a new species of *Papuanatula* from another part of New Guinea. Presently, the genus *Papuanatula* is known to have a disjunct distribution with one species on Sulawesi Island, 24 species on New Guinea, and one species on New Britain (Demoulin 1969; Lugo-Ortiz and McCafferty 1999; Kaltenbach et al. 2025). This study brings the total number of known *Papuanatula* species to 33.

The mayfly fauna of New Guinea, the second largest island of the world after Greenland, is astonishingly poor in number of families and genera. Only five of ~40 families and 13 of ~460 genera worldwide are present (Jacobus et al. 2019; Kluge 2025). From Baetidae, the most diverse family with nearly one third of all mayfly species, only five of ~115 genera have been discovered so far in New Guinea (*Centroptella* Braasch & Soldán, 1980, *Cloeon* Leach, 1815, *Labiobaetis* Novikova & Kluge, 1987, *Mystaxiops* McCafferty & Sun, 2005, and *Papuanatula* Lugo-Ortiz & McCafferty, 1999). However, two of these genera were able to develop a remarkable diversity, *Labiobaetis* with 43 species (Lugo-Ortiz et al. 1999; Kaltenbach and Gattolliat 2018, 2021; Kaltenbach et al. 2021, 2023), and *Papuanatula* with now 33 species (Kaltenbach et al. 2025). A second example of megadiversity in New Guinea mayflies is the family Leptophlebiidae, based on the tribe Thraulini (Sartori and Salles 2025). Given the results of repeated collection efforts in Batanta, it is likely that many more species of *Papuanatula* will be discovered in mainland New Guinea and nearby islands in the near future.

## ﻿Materials and methods

Most material was collected by one of us (TiKo) on the island of Batanta (Indonesia, West Papua) together with colleagues and a local team during several trips from 2014–2025. The larvae were collected by kick-sampling and preserved in 70%–96% ethanol, winged stages were attracted by light (Xenon HDI bulb, 35 Watt, 6000 Kelvin) in or close to the riverbed and also preserved in 70%–96% ethanol. Later, assignment of winged stages to a species was done by molecular analysis of COI. Eggs of one species were extracted from female subimagos.

The dissection of specimens was done in Cellosolve (2-Ethoxyethanol) with subsequent mounting on slides with Euparal liquid, using an Olympus SZX7 stereomicroscope.

DNA of part of the specimens was extracted using non-destructive methods allowing subsequent morphological analysis (see Vuataz et al. 2011 for details). We amplified a 658 bp fragment of the mitochondrial gene cytochrome oxidase subunit 1 (COI) using the primers LCO 1490 and HCO 2198 (Folmer et al. 1994). Sequencing was done with Sanger’s method (Sanger et al. 1977). The genetic variability between specimens was estimated using Kimura-2-parameter distances (K2P, Kimura 1980), calculated with the program MEGA 11 (Tamura et al. 2021, http://www.megasoftware.net). The GenBank accession numbers are given in Table 1.

**Table 1.** Sequenced specimens.

Species	Specimen voucher catalogue #	Stage	GPS coordinates	GenBank # (COI)	GenSeq Nomenclature
*P. batantaraja* sp. nov.	GBIFCH00975888	L	00°52'16"S, 130°37'46"E	PX098278	genseq-2 COI
*P. batanlenos* sp. nov.	GBIFCH00975880	L	00°52'31"S, 130°37'58"E	PX098287	genseq-2 COI
	GBIFCH00975879	L	00°52'31"S, 130°37'58"E	PX098286	genseq-2 COI
	GBIFCH00975875	L	00°54'21"S, 130°38'32"E	PX098285	genseq-2 COI
	GBIFCH00975864	SI	00°52'31"S, 130°37'58"E	PX098284	genseq-2 COI
	GBIFCH00975967	I	00°52'49"S, 130°38'05"E	PX098279	genseq-2 COI
	GBIFCH00975966	SI	00°52'49"S, 130°38'05"E	PX098280	genseq-2 COI
	GBIFCH00975969	SI	00°53'03"S, 130°38'13"E	PX098281	genseq-2 COI
	GBIFCH00975962	SI	00°53'22"S, 130°39'07"E	PX098283	genseq-2 COI
	GBIFCH00975960	SI	00°53'07"S, 130°38'59"E	PX098282	genseq-2 COI
*P. longabranchias* sp. nov.	GBIFCH00975884	L	00°52'31"S, 130°37'58"E	PX098297	genseq-2 COI
	GBIFCH00975811	I	00°53'03"S, 130°38'13"E	PX098288	genseq-2 COI
	GBIFCH00975940	I	00°53'03"S, 130°38'13"E	PX098289	genseq-2 COI
	GBIFCH00975943	SI	00°53'03"S, 130°38'13"E	PX098291	genseq-2 COI
	GBIFCH00975942	SI	00°53'03"S, 130°38'13"E	PX098290	genseq-2 COI
	GBIFCH00975954	SI	00°53'43"S, 130°36'39"E	PX098296	genseq-2 COI
	GBIFCH00975953	SI	00°53'43"S, 130°36'39"E	PX098295	genseq-2 COI
	GBIFCH00975951	SI	00°52'49"S, 130°38'05"E	PX098294	genseq-2 COI
	GBIFCH00975950	SI	00°52'49"S, 130°38'05"E	PX098293	genseq-2 COI
	GBIFCH00975946	SI	00°53'43"S, 130°36'39"E	PX098292	genseq-2 COI
*P. arfak* sp. nov.	GBIFCH00975788	L	01°06'35"S, 133°56'51"E	PX098298	genseq-2 COI
*P. cukiclara* sp. nov.	GBIFCH01582008	L	00°53'03"S, 130°38'13"E	PX098299	genseq-2 COI
	GBIFCH01582009	L	00°52'27"S, 130°37'52"E	PX098300	genseq-2 COI
	GBIFCH01582010	L	00°52'27"S, 130°37'52"E	PX098301	genseq-2 COI

Photographs of larvae in toto were taken using a Canon EOS 6D camera and processed with the programs Adobe Photoshop Lightroom (http://www.adobe.com) and Helicon Focus v. 5.3 (http://www.heliconsoft.com). Pictures of larval, subimaginal and imaginal structures were taken with an Olympus SC 50 camera on an Olympus BX43 microscope, processed with the program Olympus Cell Sense v. 3.2., and in addition with a Keyence Photomicroscope. SEM pictures were taken using a FEI Quanta FEC 250 electron microscope (Thermo Fisher). Photographs were subsequently enhanced with Adobe Photoshop Elements 13.

The distribution map was created with the program SimpleMappr (Shorthouse 2010).

The dichotomous keys were elaborated with the support of the program DKey v. 1.3.0 (http://drawwing.org/dkey, Tofilski 2018).

The terminology follows Kluge (2004). The term “blank” is used to describe an unpigmented area of cuticle (Kluge et al. 2023). The term “posterior seta/setae” is used for long setae in posterior position of the claw (approximately opposite to the distalmost denticle), as proposed by Kluge and Novikova (2014). The term “microlepides“ is used according to Kluge (2022).

### ﻿Abbreviations

**MM** Mátra Museum of the Hungarian Natural History Museum of the Hungarian National Museum Public Collection Centre, Gyöngyös (Hungary);

**MZB** Museum Zoologicum Bogoriense (Indonesia);

**MZL** Naturéum, Muséum cantonal des Sciences Naturelles, Lausanne (Switzerland).

## ﻿Results

### ﻿Subgenus Papuanatula

Taxon classificationAnimaliaEphemeropteraBaetidae

7932E497-9B92-5620-A02E-574FA696DA9C

Figs 1, 2, 3, 4, 5, 6, 7, 8, 9, 10, 11, 12, 13, 14, 15, 16, 17, 18, 19

#### Diagnosis

**(larval characters; according to Kaltenbach et al. 2025: 166).** Antennal flagellum distally with brown dots; labrum wide, widest in medial area, dorsally with submarginal row of long, feathered setae (Fig. 2b); both mandibles with incisor strongly elongated, blade-like (Fig. 7c, d; full-length incisors present only at the beginning of each instar, mostly worn at the end of instar); labial palp without clear distomedial projection at segment II (Fig. 2e); outer side of femur usually with single regular row of long, hair-like setae bearing numerous fine, short branches on all sides (Fig. 8a, b); anterior side of tibia usually with regular row of setae similar to that on femur (Fig. 8a).

##### ﻿*Papuanatula bessa* species group (Kaltenbach et al. 2025)

**Figure 1.**
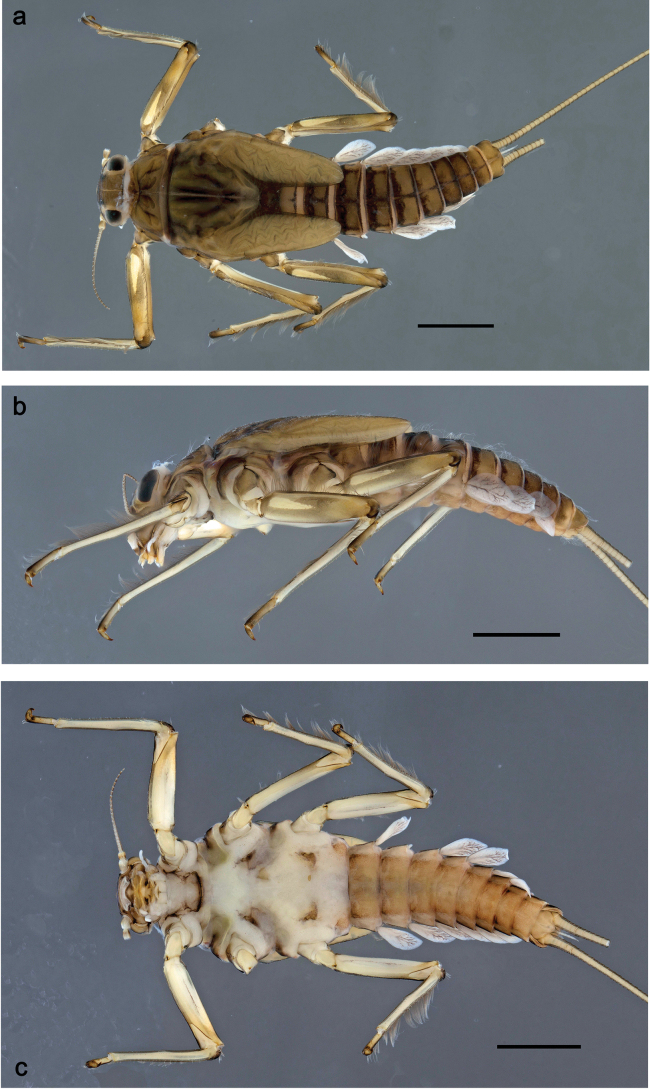
Papuanatula (Papuanatula) arfak sp. nov., larva, habitus. a. Dorsal view; b. Lateral view; c. Ventral view. Scale bars: 1 mm.

**Diagnosis (larval characters).** Body dorsally with irregular row of long, fine, soft setae on midline (Figs 1b, 5a); abdominal terga partially with paired, medioposterior humps or elevations (Kaltenbach et al. 2025: fig. 5a–e).

### ﻿ Papuanatula (Papuanatula) arfak

Taxon classificationAnimaliaEphemeropteraBaetidae

Kaltenbach, Kovács & Gattolliat sp. nov.

79AAA1BC-D8D8-5978-8D23-4314421AF9E2

https://zoobank.org/AC95E18D-EFBD-4CE1-B3C7-23F9F0DFBC7C

Figs 1, 2, 3, 4, 5

#### Type material.

***Holotype*.** Indonesia • larva; New Guinea, Papua Prov., Riv. Je, Loc. Arfak, E of Amber village; 01°06'35"S, 133°56'51"E; 1200 m; 16.vi.2016; leg. B. Sumoked and M. Balke; (BH68); on slide; GBIFCH00592543; MZB. ***Paratypes*.** 24 larvae; same data as holotype; 4 on slides; GBIFCH00975788, GBIFCH00975789, GBIFCH00592534, GBIFCH00592540, GBIFCH01221764; MZL; 20 in alcohol; GBIFCH00975790, GBIFCH00976060, GBIFCH00976063, GBIFCH00976111; MZL.

#### Diagnosis.

**Larva**. The following combination of characters distinguishes *P. arfak* sp. nov. from other species of *Papuanatula* s. str.: body dorsally with irregular row of long, fine, simple setae along midline; abdominal terga without distinct protuberances; abdominal terga I–VI with hypodermal wide dark brown transverse band close to anterior margin, I–IX with medial narrow, dark brown, longitudinal streak; femur proximally with wedge-shaped blank; abdominal terga with triangular, apically rounded denticles on posterior margin; small scattered scales on abdominal terga oblong, striated, apically denticulate; paracercus with nine segments.

#### Description.

**Figure 2.**
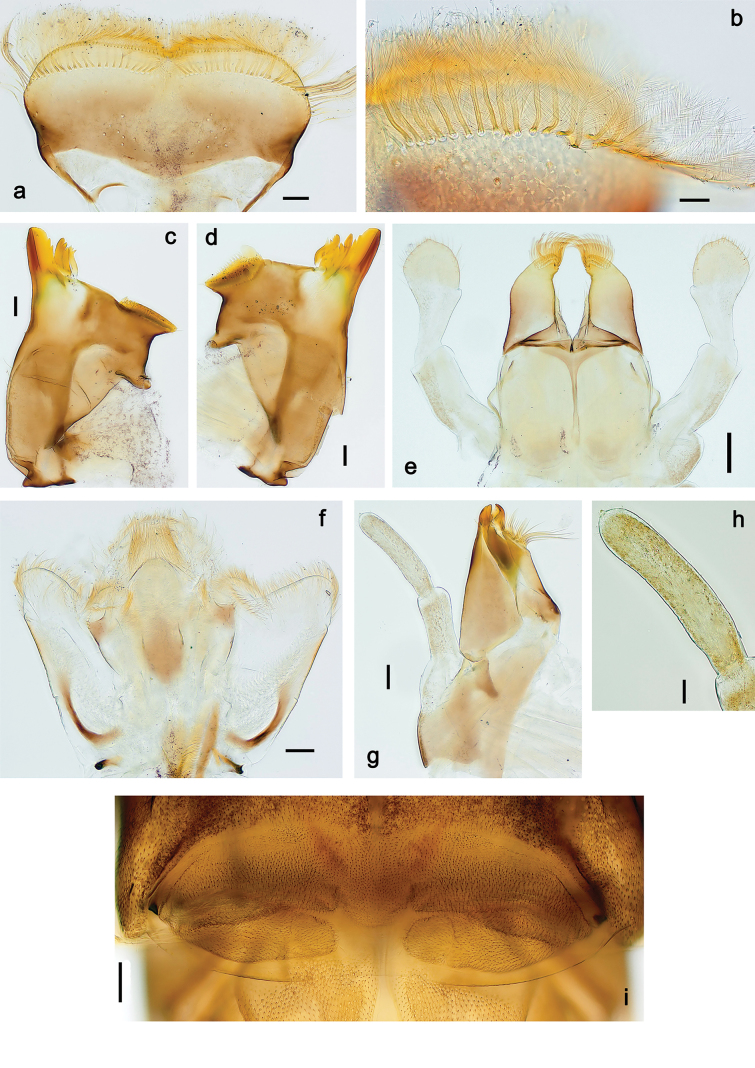
Papuanatula (Papuanatula) arfak sp. nov., larva. a. Labrum; b. Labrum, submarginal arc of setae; c. Right mandible; d. Left mandible; e. Labium; f. Hypopharynx and superlinguae; g. Maxilla; h. Maxillary palp; i. Developing subimaginal gonostyli. Scale bars: 50 µm (e), 20 µm (a, c, d, f, g, i),10 µm (b, h).

**Figure 3.**
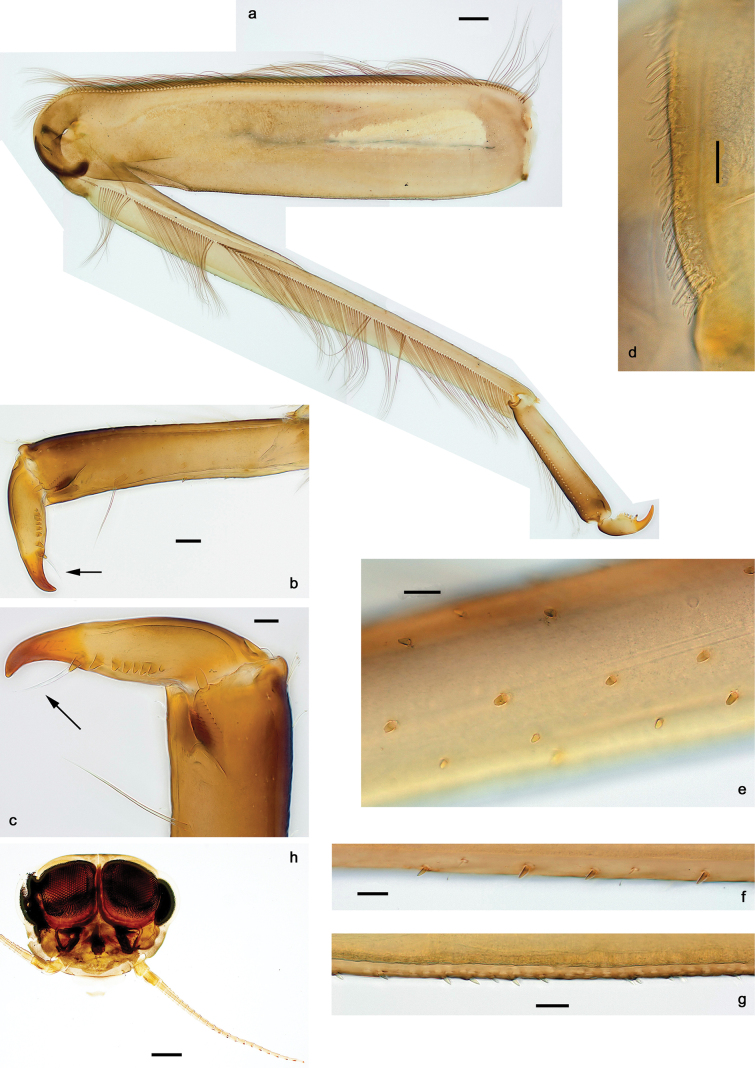
Papuanatula (Papuanatula) arfak sp. nov., larva. a. Middle leg; b. Fore tarsus; c. Fore claw; d. Fore femur, posterior apex; e. Fore tibia, posterior surface; f. Middle tibia, inner margin; g. Fore femur, ventral margin; h. Head, mature male larva. Scale bars: 100 µm (h), 50 µm (a), 20 µm (b), 10 µm (c–g).

**Figure 4.**
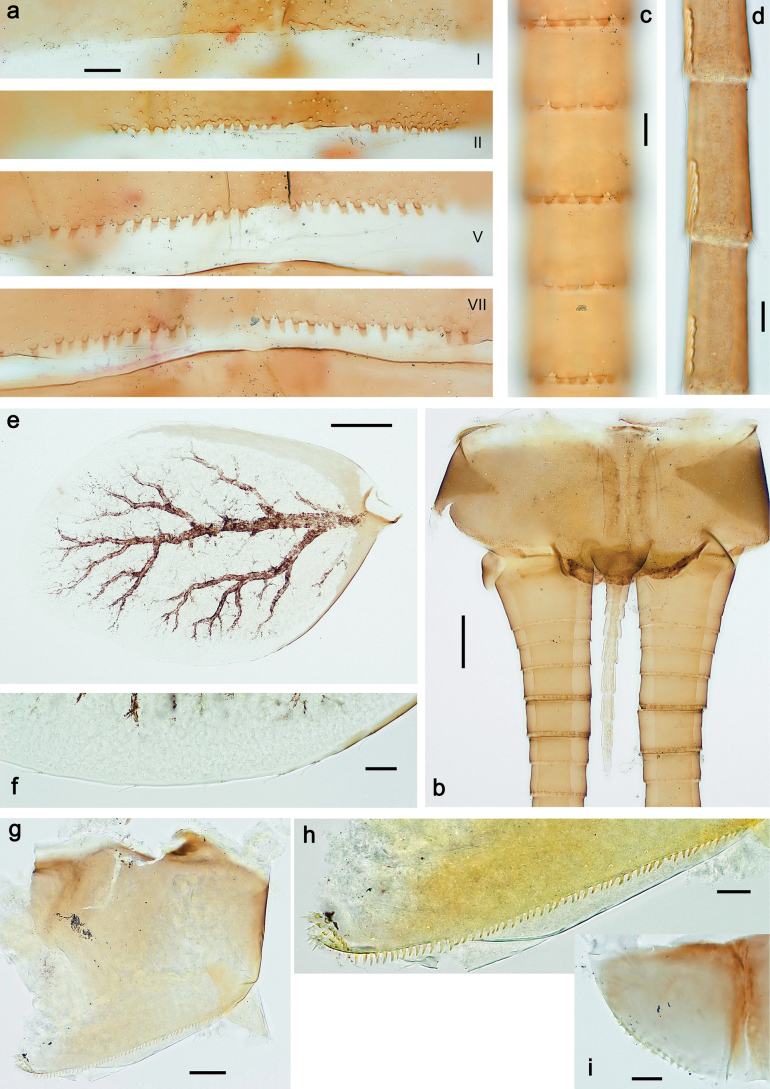
Papuanatula (Papuanatula) arfak sp. nov., larva. a. Abdominal terga, posterior margins; b. Paracercus; c. Cercus, basal part; d. Cercus, distal part; e, f. Tergalius IV; g–i. Paraproct. Scale bars: 50 µm (h), 20 µm (a, b, g), 10 µm (c, d, f, h, i).

**Figure 5.**
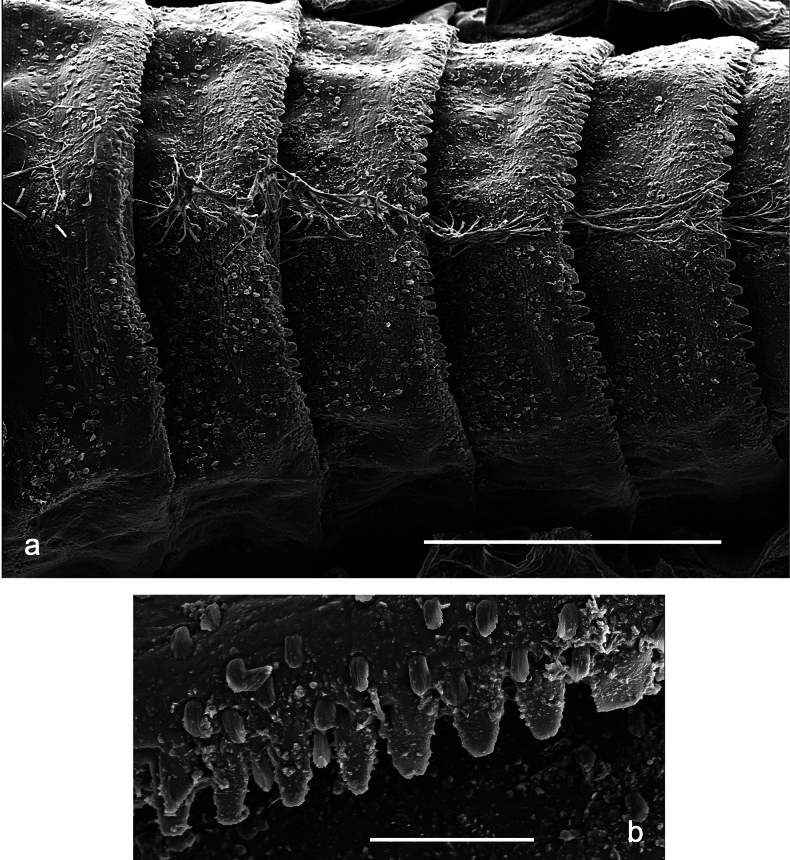
Papuanatula (Papuanatula) arfak sp. nov., larva, SEM pictures. a. Abdominal terga III–VII; b. Abdominal tergum VII, posterior margin. Scale bars: 300 µm (a), 40 µm (b).

**Larva** (Figs 1–5). Body length 4.5–5.7 mm, cerci much longer than body length (~ 1.4×).

***Cuticular coloration*** (Figs 1a–c, 3a). Head, thorax and abdomen dorsally brown; thorax with indistinct, complex pattern. Femur proximally with wedge-shaped blank, surrounded by grey area, distally yellow-brown to grey-brown; tibia grey; tarsus grey-brown, distally darker. Head, thorax and abdominal segment I ventrally ecru, protuberances of thoracic sterna brown; abdominal segments II–X ventrally brown, laterally paler. Cerci yellow-brown.

***Hypodermal coloration*** (Fig. 1a). Abdominal terga I–VI (VII) with wide dark brown transverse band close to anterior margin, I–IX with narrow dark brown transverse band close to posterior margin and medially narrow dark brown, longitudinal streak.

***Head*** (Figs 1b, 3h). Dorsally with irregular row of long, fine, simple setae along midline.

***Antenna*.** Length ~1.5× head length. As typical for subgenus.

***Developing turbinate eyes in last instar male larva*** (Fig. 3h) large, subquadrangular, touching each other in the middle.

***Labrum*** (Fig. 2a, b). Length 0.5× maximum width, laterally convex. Dorsal, sub-marginal arc with ~ 30 feathered setae.

***Right mandible*** (Fig. 2c). Margin between prostheca and mola straight, with row of minute denticles. Otherwise, as typical for subgenus.

***Left mandible*** (Fig. 2d). Margin between prostheca and mola straight, with row of minute denticles. Otherwise, as typical for subgenus.

***Hypopharynx*** (Fig. 2f). As typical for genus.

***Maxilla*** (Fig. 2g, h). Maxillary palp subequal in length to galea-lacinia; palp segment II slightly longer than segment I. Otherwise, as typical for the genus.

***Labium*** (Fig. 2e). Paraglossa dorsally with two spine-like setae near inner, distolateral margin. Labial palp with segment I subequal in length to segments II and III combined. Segment II with minute distomedial protuberance, dorsally with row of five or six spine-like setae near outer, distolateral margin. Segment III slightly pentagonal, pointed; 0.8× length of segment II. Otherwise, as typical for the genus.

***Thorax*. *Sterna*** (Fig. 1c). With small protuberances on sides of prosternum and close to openings of mesothoracic and metathoracic sternal apodemes.

***Terga*** (Fig. 1b) without protuberance; with irregular row of long, fine, simple setae along midline.

***Legs*** (Fig. 3a–g). Ratio of leg segments: fore leg 0.9:1.0:0.3:0.2, middle leg 1.0:1.0:0.3:0.2 and hind leg 1.1:1.0:0.3:0.2. ***Femur*.** Length ~ 4× maximum width. ***Claw*** with one row of 5–8 denticles and one posterior setae. Otherwise, as typical for subgenus.

***Abdomen*. *Terga*** (Figs 1b, 4a, 5a, b) with irregular row of long, fine, simple setae along midline. Terga without protuberances, terga I–IV with slight, paired medioposterior elevations. Posterior margin of terga: I smooth, without denticles, II–IX with triangular, partly apically rounded denticles, becoming longer toward end of abdomen. Surface with scattered small, oblong, striated, apically serrate scales.

***Tergalii*** (Fig. 4e, f). Present on terga II–VII. Broad ovoid, tracheation well developed and pigmented; margin smooth, with short, fine, simple setae. Tergalius II nearly reaching end of tergum IV, tergalius IV as long as length of terga V and VI combined, tergalius VII nearly reaching end of tergum IX.

***Paraproct*** (Fig. 4g–i). Posterior margin with prolongation and dense row of minute denticles.

***Caudalii*** (Fig. 4b–d). Cerci apart from basal part with 1–6 swimming setae per segment, increasing toward distal part. Paracercus with nine segments.

***Pose of subimaginal gonostyli under larval cuticle*** (Fig. 2i) as typical for the subgenus.

**Subimago.** Unknown.

**Imago.** Unknown.

**Egg.** Unknown.

#### Biological aspects.

The species was found at an altitude of 1200 m, together with *Papuanatula dumspinae* Kaltenbach, Kluge & Gattolliat, 2025, *Papuanatula paratuber* Kaltenbach, Kluge & Gattolliat, 2025, *Papuanatula epituber* Kaltenbach, Kluge & Gattolliat, 2025, and *Papuanatula pilosa* Kaltenbach, Kluge & Gattolliat, 2025.

#### Etymology.

The species name refers to the locality Arfak, the type locality of the species.

#### Distribution.

New Guinea (Fig. 30).

##### ﻿Key to the species of *P. bessa* species group from New Guinea (larvae)

Based on Kaltenbach et al. 2025.

**Table:** 

1	Metanotum and abdominal terga I–III or I–V with medioposterior broad, paired humps (Kaltenbach et al. 2025: fig. 56c), may be poorly developed (Kaltenbach et al. 2025: fig. 47a)	**2**
–	Metanotum and abdominal terga without paired humps	**4**
2(1)	Metanotum and abdominal terga I–V with medioposterior, paired humps; femur with wedge-shaped blank in proximal area; claw with one posterior seta	**3**
–	Metanotum and abdominal terga I–III with medioposterior, paired humps poorly developed; femur without wedge shaped blank; claw with 1–3 posterior setae	***P. epibessa***
3(2)	Abdominal terga II–IV dark brown with brighter, oblong marking	***P. bessa***
–	Abdominal terga II–VI with paired, semicircular, dark brown markings	***P. parabessa***
4(1)	Paracercus vestigial (max. 2 segments); claw with 3 or 4 posterior setae	***P. pluresetae***
–	Paracercus with 7–9 segments; claw with 1 posterior seta	**5**
5(4)	Abdominal terga II–IX with long, triangular, pointed denticles on posterior margins	**6**
–	Abdominal terga II–IX with triangular, apically rounded denticles on posterior margins	**7**
6(5)	Abdominal terga V, VI and X much brighter than other terga; apical setal rows of paraglossae curved; triangular, pointed denticles on posterior margins of abdominal terga long and narrow	***P. dumspinae***
–	Abdomen with rather uniform colour; apical setal rows of paraglossae straight; triangular, pointed denticles on posterior margins of abdominal terga of different length	***P. cyclopomontana***
7(5)	Femur with clearly outlined wedge-shaped blank; abdominal terga dark brown, no terga distinctly brighter or terga I, V, VI, and X brighter; small scales on abdominal terga roundish or oblong, striated	**8**
–	Femur with wedge-shaped blank, overlaid with scattered brownish colour; abdominal terga dark brown, terga V, VI, and X much brighter; small scales on abdominal terga elongate, slightly trapezoid, striated	***P. balkei***
8(7)	Abdominal terga brown, terga III and IV with grate-like marking (dark brown longitudinal streaks laterally and medially); small scales on abdominal terga oblong, apically serrate	***P. arfak* sp. nov.**
–	Abdominal terga brown, terga I, V, VI and X brighter; small scales on abdominal segments roundish	***P. obscura***

##### ﻿*Papuanatula copis* species group (Kaltenbach et al. 2025)

**Figure 6.**
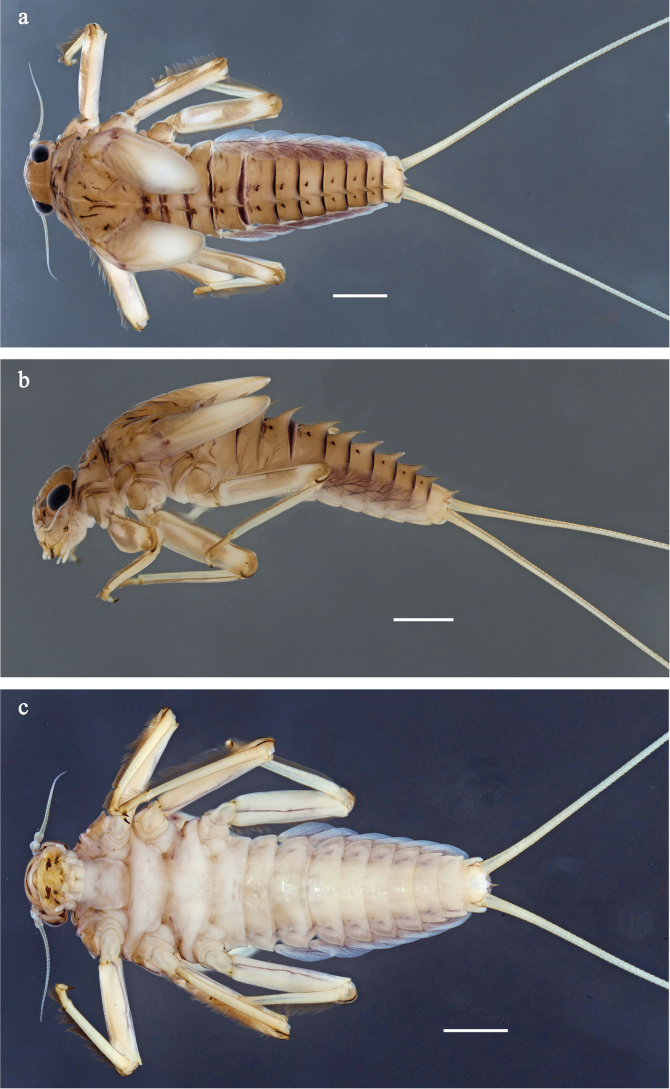
Papuanatula (Papuanatula) batantaraja sp. nov., larva, habitus. a. Dorsal view; b. Lateral view; c. Ventral view. Scale bars: 1 mm.

**Diagnosis (larval characters).** Body dorsally without row of setae on midline; abdomen and sometimes also thorax dorsally with unpaired, conspicuous protuberances (Figs 6b, 8e).

### ﻿ Papuanatula (Papuanatula) batantaraja

Taxon classificationAnimaliaEphemeropteraBaetidae

Kovács, Kaltenbach & Gattolliat sp. nov.

13E61C5A-A69F-58A6-B76B-6E98436A730A

https://zoobank.org/EA683BE4-0A2F-44C0-AD21-45E38B61BE4F

Figs 6, 7, 8

#### Type material.

***Holotype*.** Indonesia • larva; West Papua, Batanta Island, right side stream of Kalijakut River; 00°52'16"S, 130°37'45.5"E; 545 m; 10.ii.2024; leg. T. Kovács and R. Sauyai; on slides; GBIFCH00975887, GBIFCH01221819; 2024-13.b, EPHTYP-1; MM. ***Paratypes*.** 4 larvae; same data as holotype; 2 on slides; GBIFCH00975888, GBIFCH01221825, GBIFCH01221826; 2 in alcohol; GBIFCH00975886; MZL • 13 larvae; partly same data as holotype; 21.ii.2025; leg. T. Kovács; 2 in alcohol; GBIFCH01582004; MZL; 11 in alcohol; 2025-19, EPHTYP-2; MM • 1 larva; West Papua, Batanta Island, right side stream of Kalijakut River, side spring area; 00°52'27"S, 130°37'51"E; 432 m; 20.ii.2025; leg. T. Kovács; in alcohol; 2025-18.b, EPHTYP-3; MM.

#### Diagnosis.

**Larva**. The following combination of characters distinguishes *P. batantaraja* sp. nov. from other species of *Papuanatula* s. str.: large species (> 7 mm body length); body dorsally without row of long, fine, simple setae along midline; fore protoptera posteromedially with pair of minute, broad protuberances and one minute, rather pointed protuberance between them; metanotum and abdominal terga I–X medially with conspicuous, long, pointed protuberance, slightly bent posteriad; left mandible without subtriangular process; femur anteriorly with angulate blank in basal ½; transparent, semicircular scales on anterior surface of femur and dorsal surface of abdominal terga; paracercus vestigial.

#### Description.

**Figure 7.**
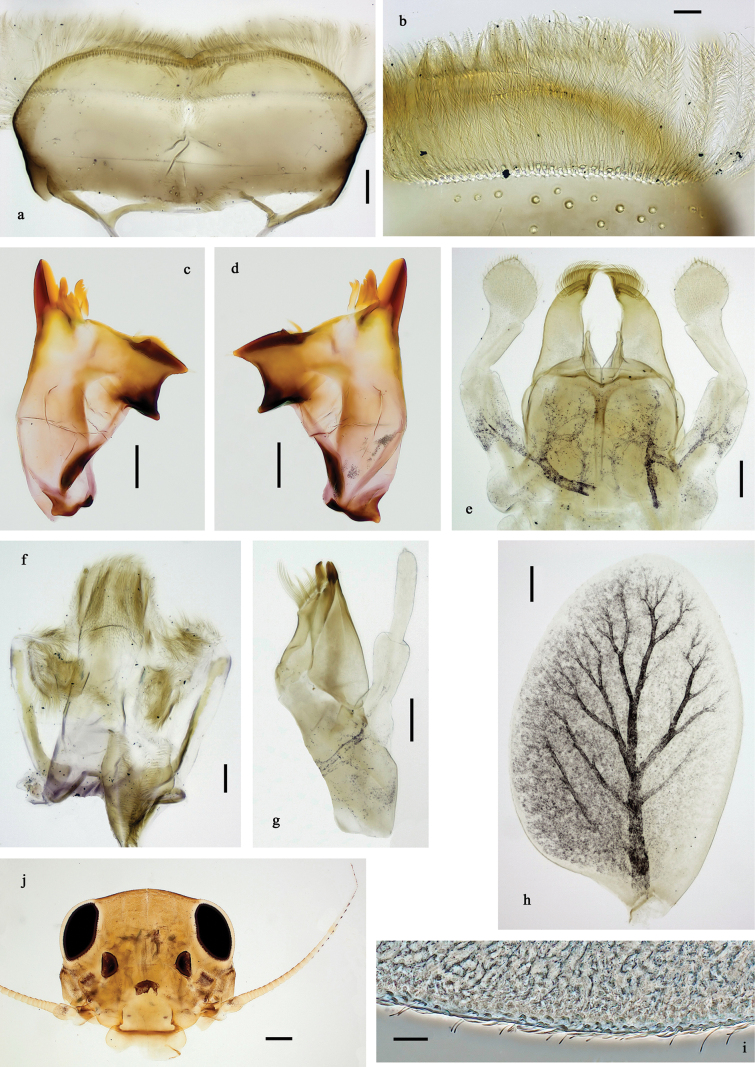
Papuanatula (Papuanatula) batantaraja sp. nov., larva. a. Labrum; b. Labrum, submarginal arc of setae; c. Right mandible; d. Left mandible; e. Labium; f. Hypopharynx and superlinguae; g. Maxilla; h, i. Tergalius IV; j. Head. Scale bars: 100 µm (j), 20 µm (a, c–h),10 µm (b, i).

**Figure 8.**
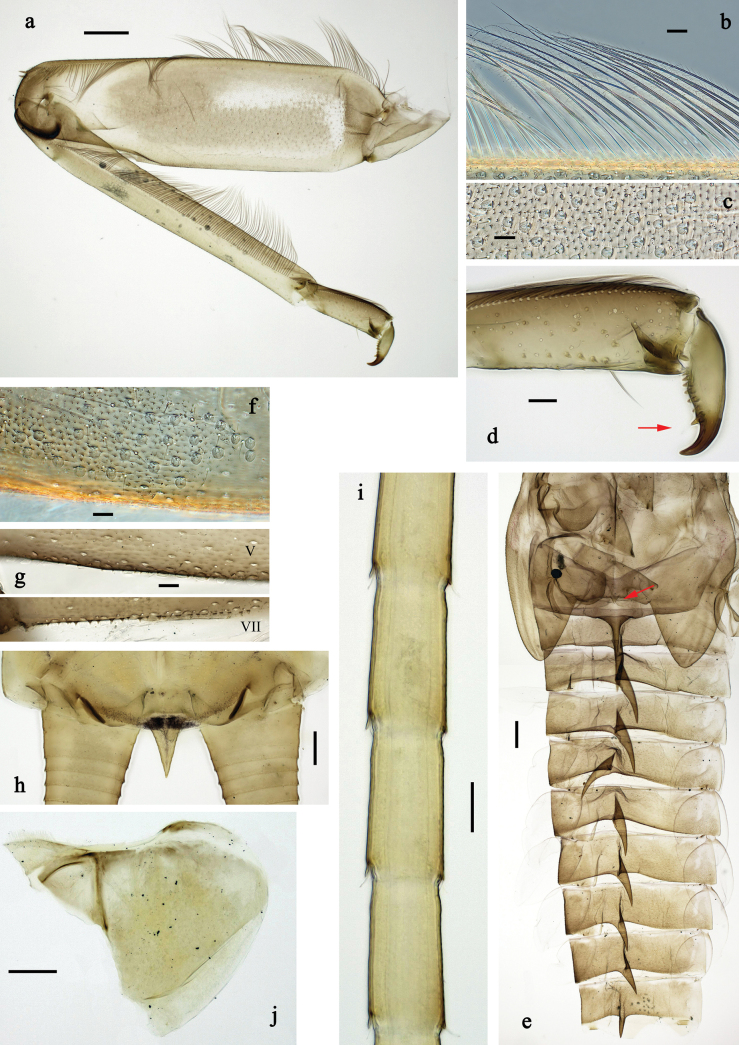
Papuanatula (Papuanatula) batantaraja sp. nov., larva. a. Hind leg; b. Hind femur, outer margin; c. Hind femur, surface; d. Hind tarsus, claw (arrow: posterior seta); e. Abdomen (arrow: protuberance on fore protoptera); f. Abdominal tergum V, surface; g. Abdominal terga; h. Paracercus; i. Cercus; j. Paraproct. Scale bars: 100 µm (e), 50 µm (a), 20 µm (b, d, h, j), 10 µm (c, f, g, i).

**Larva** (Figs 6–8). Body length 7.0–7.5 mm, largest *Papuanatula* species known so far. Cerci ~2× as long as body length.

***Cuticular coloration*** (Figs 6a–c, 8e). Head, thorax and abdomen dorsally uniform grey-brown. Head, thorax and abdomen ventrally beige. Legs yellow-brown, femur basally with angulate blank, apically blank. Caudalii yellow-brown.

***Hypodermal coloration*** (Fig. 6a, b). Thorax dorsally with few blackish markings. Abdominal terga with narrow dark brown to blackish transverse band along posterior margins; sigillae blackish.

***Head*. *Antenna*** (Fig. 7j). Length ~1.5× head length. Flagellum distally with brown dots.

***Developing turbinate eyes in last instar male larva*** unknown.

***Labrum*** (Fig. 7a, b). Very wide, length 0.5× maximum width, laterally angulate. Dorsal, sub-marginal arc with >40 densely articulated, feathered setae.

***Right mandible*** (Fig. 7d). Margin between prostheca and mola straight, smooth. Otherwise, as typical for the subgenus.

***Left mandible*** (Fig. 7c). Margin between prostheca and mola straight, smooth; subtriangular process not developed. Otherwise, as typical for the subgenus.

***Hypopharynx*** (Fig. 7f). Apical tuft of spine-like setae laterally denser, giving the impression of a pair of tufts. Otherwise, as typical for the genus.

***Maxilla*** (Fig. 7g). Maxillary palp slightly longer than galea-lacinia; palp segment II approx. as long as segment I; segment I thicker than segment II. Otherwise, as typical for the genus.

***Labium*** (Fig. 7e). Paraglossa dorsally with one spine-like seta near inner, distolateral margin. Labial palp with segment I 0.9× length of segments II and III combined. Segment II with very small, rounded, distomedial protuberance, dorsally with row of five spine-like setae near outer, distolateral margin. Segment III slightly pentagonal, pointed, 0.8× length of segment II. Otherwise, as typical for the genus.

***Thorax*. *Sterna***. Protuberances not developed.

***Terga*** (Fig. 8e). Metanotum posteromedially with long, pointed protuberance, slightly bent posteriad. Fore protoptera posteromedially with pair of minute, broad protuberances and one minute, rather pointed protuberance between them.

***Legs*** (Fig. 8a–d). Ratio of leg segments: fore leg 1.1:1.0:0.3:0.1, middle leg 1.0:1.0:0.3:0.1 and hind leg 1.1:1.0:0.3:0.1. ***Femur***. Length ~ 3× maximum width. Anterior surface with transparent, semicircular scales. ***Claw*** with one row of 7–9 denticles, apical denticle larger than other ones, and one or two posterior setae, and one or two short, reduced setae opposite to posterior setae. Otherwise, as typical for the subgenus.

***Abdomen*. *Terga*** (Figs 6a, b, 8e–g). Abdominal terga I–X posteromedially with conspicuous, long, pointed protuberance, slightly bent posteriad; Posterior margin of terga: I–IX with minute, pointed denticles, slightly increasing in size toward IX. Surface with scattered transparent, semicircular scales.

***Tergalii*** (Fig. 7h, i). Present on terga II–VII. Broad oblique ovoid; tracheation strongly developed; with grey pigmentation, especially in anal ½; margins smooth, with many short, fine, simple setae. Tergalius II as long as abdominal terga III and IV combined, tergalius IV as long as terga V, VI and ⅓ VII combined, tergalius VII reaching beginning of tergum X.

***Paraproct*** (Fig. 8j). Posterior margin expanded, smooth.

***Caudalii*** (Fig. 8h, i) Cerci without swimming setae. Paracercus vestigial.

***Pose of subimaginal gonostyli under larval cuticle*.** Unknown.

**Subimago.** Unknown.

**Imago.** Unknown.

**Egg.** Unknown.

#### Biological aspects.

The species is known from the highest watercourse of Batanta, the Kalijakut River system, on altitudes between 430 m and 545 m. The upper habitat (Fig. 29a, type locality) has volcanic bedrock, the side branch is very fast-flowing, the larvae live on the stones of the steep run. It co-occurs with larvae of *P. batanlenos* sp. nov. and *P. cukiclara* sp. nov. In the lower habitat (Fig. 29b), the species is living in fast flowing water on steep, calcareous surfaces, associated with *P. cukiclara* sp. nov.

#### Etymology.

The species name *batantaraja* refers to the Indonesian island Batanta, where the species was found, and the Indonesian word “raja” meaning king, because it is the largest species known in the genus *Papuanatula*.

#### Distribution.

New Guinea, Batanta Island (Fig. 30).

### ﻿ Papuanatula (Papuanatula) cukiclara

Taxon classificationAnimaliaEphemeropteraBaetidae

Kaltenbach, Kovács & Gattolliat sp. nov.

77142C5D-C41F-5B85-9C33-4948BF519B49

https://zoobank.org/BAA5E384-8100-45F4-8790-F4BB63B1F102

Figs 9, 10, 11

#### Type material.

***Holotype*.** Indonesia • larva; West Papua, Batanta Island, Kalijakut River; 00°52'27"S, 130°37'52"E; 420 m; 09.ii.2024; leg. T. Kovács; on slide; GBIFCH00975889; 2024-12, EPHTYP-4; MM. ***Paratypes*.** 9 larvae; partly same data as holotype; 20.ii.2025; leg. T. Kovács; 3 in alcohol; GBIFCH01582007, GBIFCH01582009, GBIFCH001582010; MZL; 6 in alcohol; 2025-18.a, EPHTYP-5; MM • 3 larvae; West Papua, Batanta Island, right side stream of Kalijakut River, side spring area; 00°52'27"S, 130°37'51"E; 432 m; 20.ii.2025; leg. T. Kovács; 1 in alcohol; GBIFCH01582006; MZL; 2 in alcohol; 2025-18.b, EPHTYP-6; MM • 4 larvae; West Papua, Batanta Island, right side stream of Kalijakut River; 00°52'15"S, 130°37'45"E; 545 m; 21.ii.2025; leg. T. Kovács; 1 on slide; GBIFCH01221838; 1 in alcohol; GBIFCH01582005; MZL; 2 in alcohol; 2025-19, EPHTYP-7; MM • 3 larvae; West Papua, Batanta Island, Kalijakut River; 00°53'03"S, 130°38'13"E; 182 m; 19.ii.2025; leg. T. Kovács; 1 on slide; GBIFCH01221837; 1 in alcohol; GBIFCH01582008; MZL; 1 in alcohol; 2025-17, EPHTYP-8; MM.

#### Diagnosis.

**Larva**. The following combination of characters distinguishes *P. cukiclara* sp. nov. from other species of *Papuanatula* s. str.: body dorsally without row of long, fine, simple setae along midline; metanotum and abdominal terga I–VIII posteromedially with conspicuous, long, pointed protuberance, slightly bent posteriad; femur anteriorly with irregular wedge-shaped blank in basal part, blank area in distal part, and blank streak along outer margin; paracercus with five or six segments.

#### Description.

**Figure 9.**
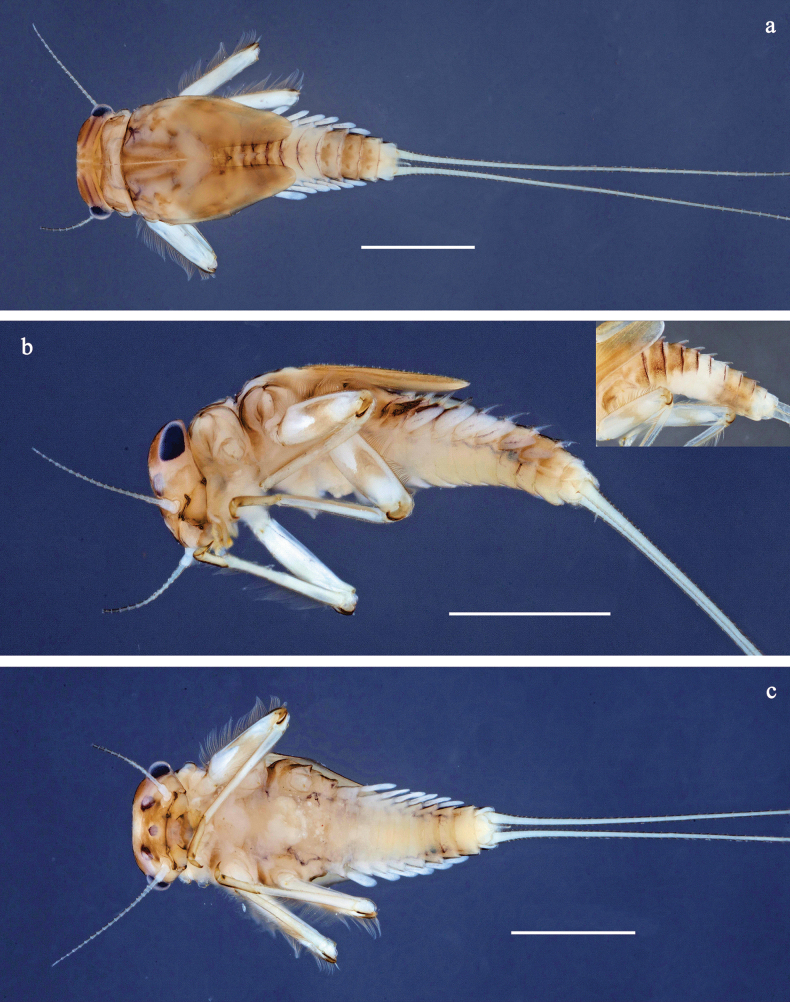
Papuanatula (Papuanatula) cukiclara sp. nov., larva, habitus. a. Dorsal view; b. Lateral view; c. Ventral view. Scale bars: 1 mm.

**Figure 10.**
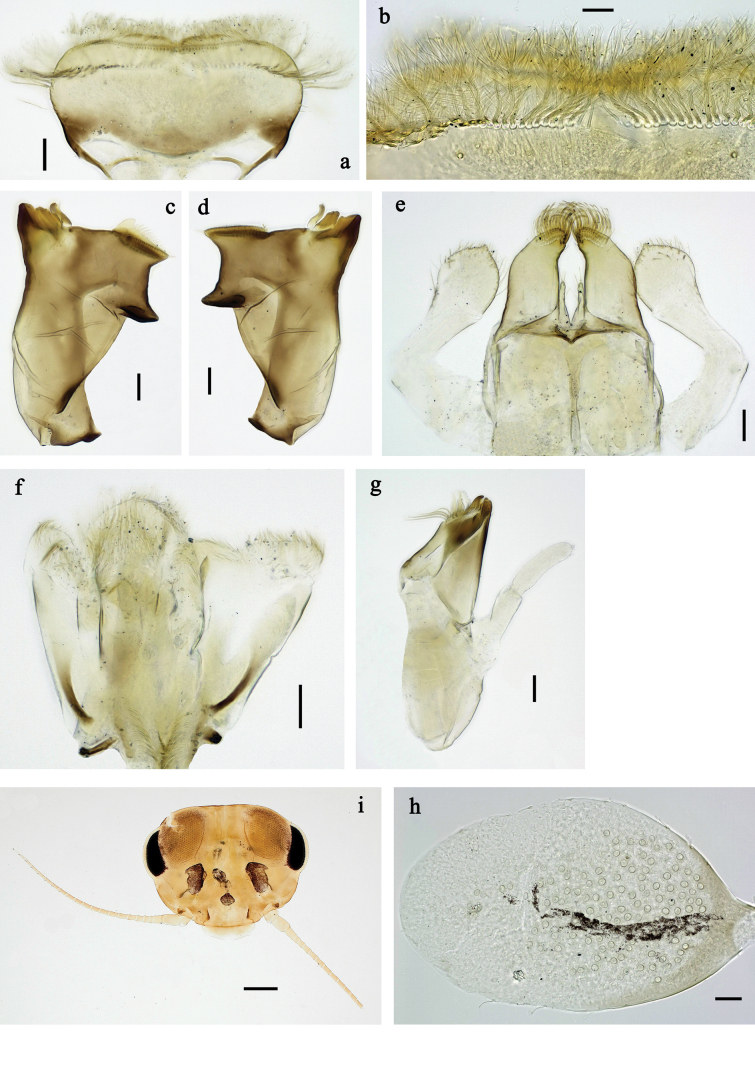
Papuanatula (Papuanatula) cukiclara sp. nov., larva. a. Labrum; b. Labrum, submarginal arc of setae; c. Left mandible; d. Right mandible; e. Labium; f. Hypopharynx and superlinguae; g. Maxilla; h. Tergalius IV; i. Head. Scale bars: 100 µm (i), 20 µm (a, c–g),10 µm (b, h).

**Figure 11.**
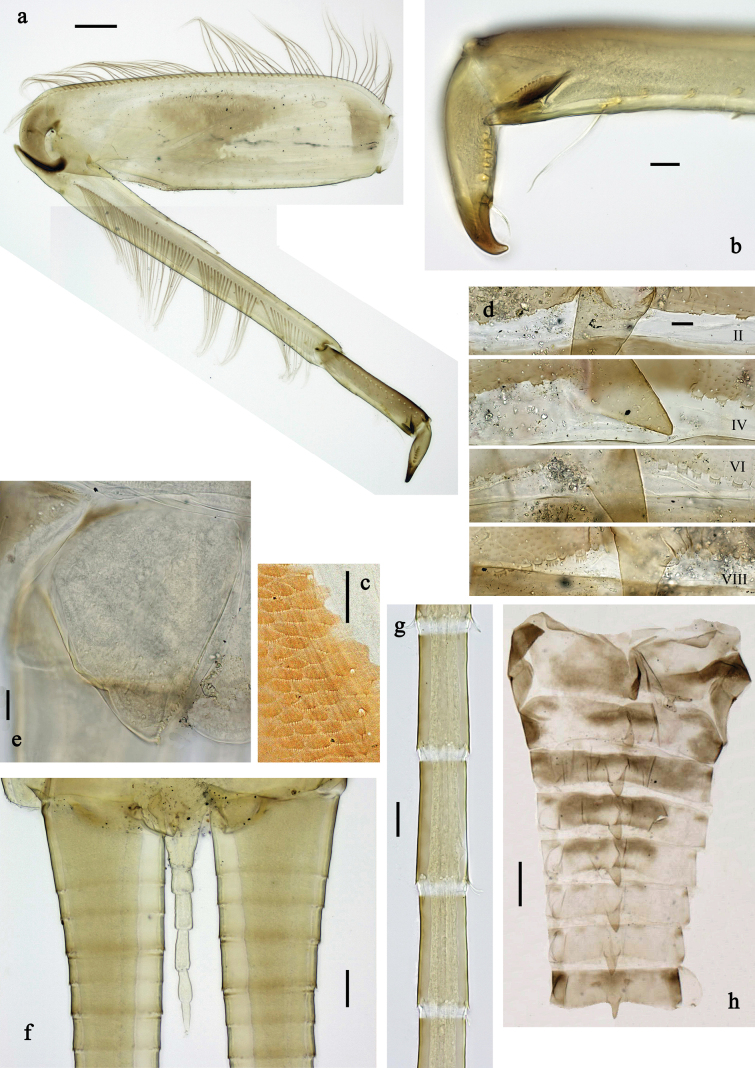
Papuanatula (Papuanatula) cukiclara sp. nov., larva. a. Hind leg; b. Fore tarsus, claw; c. Fore femur, anterior surface (brown areas); d. Abdominal terga; e. Paraproct; f. Paracercus; g. Cercus; h. Abdomen. Scale bars: 100 µm (h), 50 µm (a), 20 µm (f), 10 µm (b–e, g).

**Larva** (Figs 9–11). Body length 2.7–3.9 mm, cerci ~ 2× body length.

***Cuticular coloration*** (Figs 9a–c, 11c). Head, thorax and abdomen dorsally pale brown; thorax with indistinct pattern; abdominal terga V, VI, and X dorsally and laterally brighter, terga III and IV laterally brighter. Head, thorax and abdomen ventrally pale brown. Legs pale brown, femur anteriorly with irregular wedge-shaped blank in basal part, blank area in distal part, and blank streak along outer margin. Caudalii ecru.

***Hypodermal coloration*** (Fig. 9a, b). Abdominal terga with fine dark brown transverse band along posterior margins.

***Head*. *Antenna*** (Fig. 10i). Length ~1.5× head length. Flagellum distally with brown dots.

***Developing turbinate eyes in last instar male larva*** unknown.

***Labrum*** (Fig. 10a, b). Length ~ 0.5× maximum width, laterally convex. Dorsal, sub-marginal arc with ~ 22 feathered setae.

***Right mandible*** (Fig. 10d). Margin between prostheca and mola straight, smooth. Otherwise, as typical for the subgenus.

***Left mandible*** (Fig. 10c). Margin between prostheca and mola straight, smooth. Otherwise, as typical for the subgenus.

***Hypopharynx*** (Fig. 10f). As typical for the genus.

***Maxilla*** (Fig. 10g). Maxillary palp approx. as long as galea-lacinia; palp segment II approx. as long as segment I. Otherwise, as typical for the genus.

***Labium*** (Fig. 10e). Paraglossa dorsally with two spine-like setae near inner, distolateral margin. Labial palp with segment I 0.7× length of segments II and III combined. Segment II without distomedial protuberance, dorsally with row of four spine-like setae near outer, distolateral margin. Segment III slightly pentagonal, pointed, 0.8× length of segment II. Otherwise, as typical for the genus.

***Thorax*. *Sterna***. Protuberances not developed.

***Terga*** (Figs 9a, 11c). Metanotum posteromedially with stout, pointed protuberance.

***Legs*** (Fig. 11a–c). Ratio of leg segments: fore leg 0.9:1.0:0.3:0.2, middle leg 1.0: 1.0: 0.3: 0.2, and hind leg 1.0:1.0:0.3:0.2. ***Femur***. Length ~ 3× maximum width; surface rough, denticulate on brown areas. ***Claw*** with one row of seven or eight denticles, and one posterior seta. Otherwise, as typical for the subgenus.

***Abdomen*. *Terga*** (Figs 9a, b, 11h). Abdominal terga I–VIII posteromedially with conspicuous, long, pointed protuberance, slightly bent posteriad; Posterior margin of terga: I smooth, without spines, II–IX with short, rounded spines, apically carrying needle-like denticles.

***Tergalii*** (Fig. 10h). Present on terga II–VII. Oblique ovoid; tracheation poorly developed, pigmentation of tracheae limited to main trunk; margins smooth, with short, fine, simple setae. Tergalius II as long as abdominal terga III and IV combined, tergalius IV as long as terga V and VI combined, tergalius VII reaching middle of tergum IX.

***Paraproct*** (Fig. 11e). Posterior margin expanded, with minute denticles in distal part.

***Caudalii*** (Fig. 11f, g) Cerci without swimming setae. Paracercus with six segments.

***Pose of subimaginal gonostyli under larval cuticle*.** As typical for the subgenus (folding in “*Labiobaetis*”-type).

**Subimago.** Unknown.

**Imago.** Unknown.

**Egg.** Unknown.

#### Biological aspects.

This species is known from the Kalijakut river system (Fig. 29a, b, d type locality), occurring at elevations of 180–545 m. The larvae co-occur with *P. batanlenos* sp. nov., *P. batantaraja* sp. nov., and *P. longabranchias* sp. nov.

#### Etymology.

The species name is composed of “cuki”, meaning cute in Hungarian, and “clara”, meaning “bright” in Latin. The latter refers to the bright colour of the larva, especially on parts of the abdomen.

#### Distribution.

New Guinea, Batanta Island (Fig. 30).

### ﻿ Papuanatula (Papuanatula) cataracta

Taxon classificationAnimaliaEphemeropteraBaetidae

Kaltenbach, Kovács & Gattolliat sp. nov.

560DB812-17BB-52A5-B0B3-4CC75F8DB52D

https://zoobank.org/4039B519-414C-4439-80C2-9490D45FAADE

Figs 12, 13, 14

#### Type material.

***Holotype*.** Indonesia • larva; West Papua, Batanta Island, Warikambon Stream, upper waterfall; 00°50'25"S, 130°42'32"E; 159 m; 12.ii.2020; leg. T. Kovács, R. Horváth and P. Juhász; on slide; GBIFCH00976003; 2020-5, EPHTYP-9; MM. ***Paratypes*.** 6 larvae; same data as holotype; 3 on slides; GBIFCH00976000, GBIFCH00976001, GBIFCH00976002; 3 in alcohol; GBIFCH00975900, GBIFCH00975997, GBIFCH00975998; MZL • 27 larvae; partly same data as holotype; 04.ii.2024; leg. T. Kovács; 20 in alcohol; GBIFCH00975869, GBIFCH00975870, GBIFCH00975871, GBIFCH00975872, GBIFCH00975873; MZL; 7 in alcohol; 2024-8, EPHTYP-10; MM.

#### Diagnosis.

**Larva**. The following combination of characters distinguishes *P. cataracta* sp. nov. from other species of *Papuanatula* s. str.: body dorsally without row of long, fine, simple setae along midline; metanotum and abdominal terga I–VIII posteromedially with cone-like, pointed protuberance, longest on terga III–V, small on VII and VIII; femur anteriorly with irregular blank in basal part; anterior surface of femur and abdominal terga covered with minute, pointed spines; paracercus with five segments.

#### Description.

**Figure 12.**
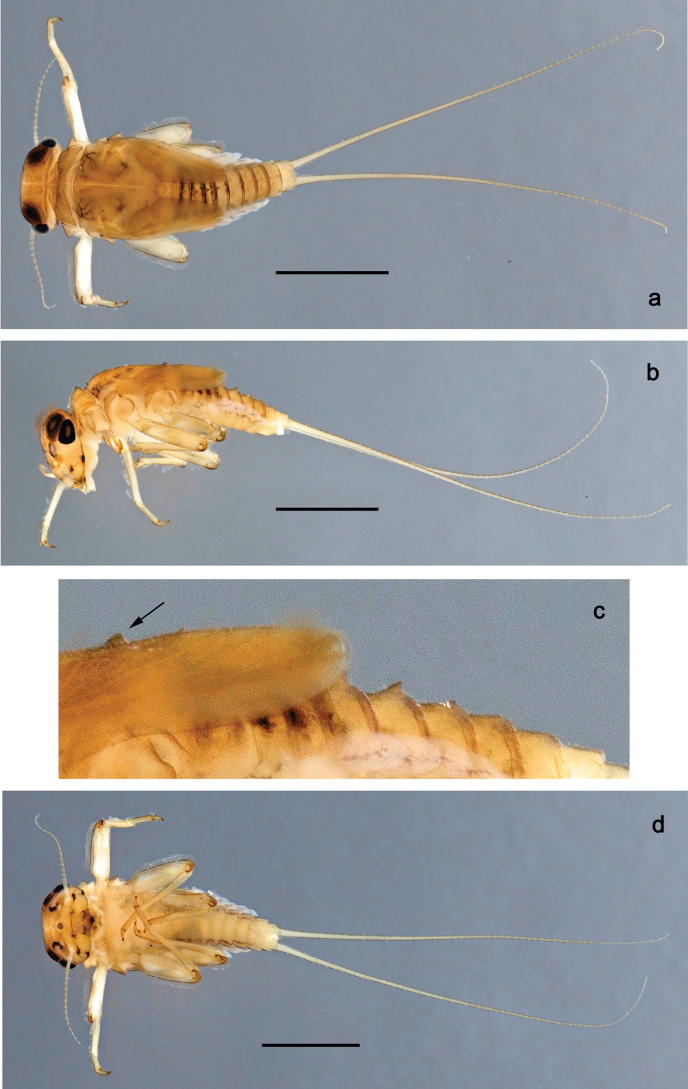
Papuanatula (Papuanatula) cataracta sp. nov., larva, habitus. a. Dorsal view; b, c. Lateral view; d. Ventral view. Scale bars: 1 mm.

**Figure 13.**
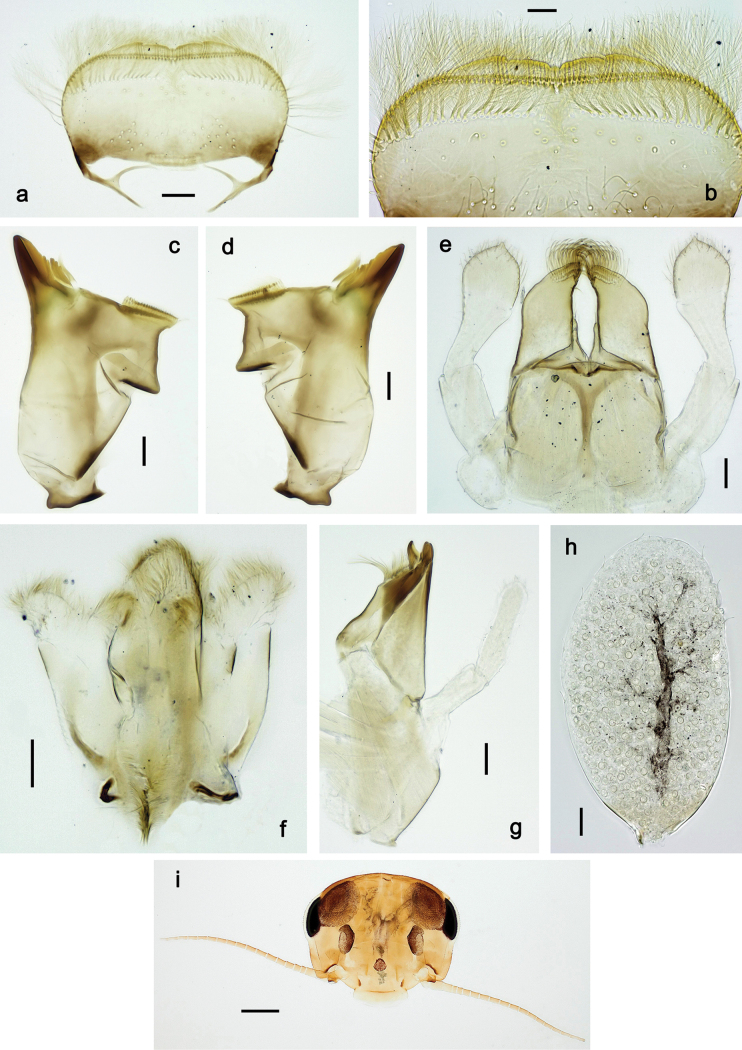
Papuanatula (Papuanatula) cataracta sp. nov., larva. a. Labrum; b. Labrum, submarginal arc of setae; c. Right mandible; d. Left mandible; e. Labium; f. Hypopharynx and superlinguae; g. Maxilla; h. Tergalius IV; i. Head. Scale bars: 100 µm (i), 20 µm (a, c–g),10 µm (b, h).

**Figure 14.**
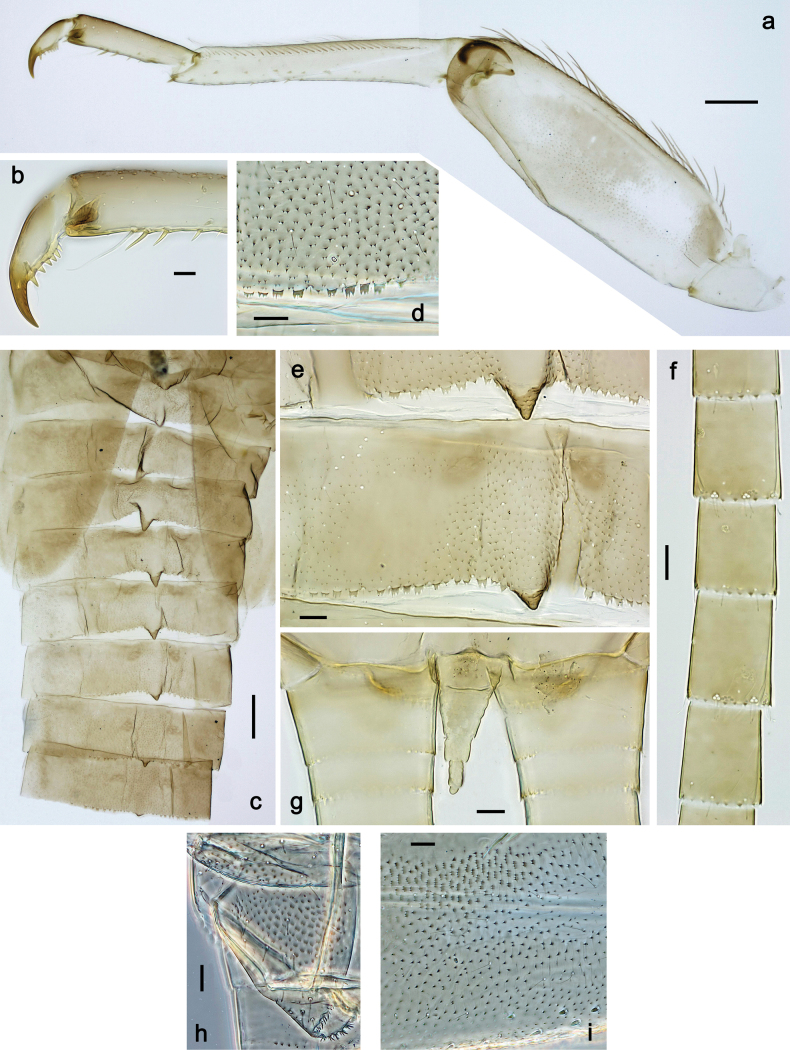
Papuanatula (Papuanatula) cataracta sp. nov., larva. a. Hind leg; b. Hind tarsus and claw; c. Abdomen; d. Abdominal tergum V; e. Abdominal terga V and VI; f. Cercus; g. Paracercus; h. Paraproct; i. Hind femur, surface. Scale bars: 50 µm (a, c), 10 µm (b, d–i).

**Larva** (Figs 12–14). Body length 2.5–3.4 mm, cerci ~ 1.6× as long as body length.

***Cuticular coloration*** (Figs 12a–d, 14c). Head, thorax and abdomen dorsally pale brown. Head, thorax and abdomen ventrally pale brown. Legs pale brown, femur anteriorly with irregular blank areas in basal and distal part. Caudalii pale brown.

***Hypodermal coloration*** (Fig. 12a). Abdominal terga with narrow dark brown, transverse band along posterior margins.

***Head*. *Antenna*** (Fig. 13i). Length ~1.5× head length. Flagellum distally without brown dots.

***Developing turbinate eyes in last instar male larva*** (Fig. 13i) rather small, ovoid, widely spaced.

***Labrum*** (Fig. 13a, b). Length ~ 0.5× maximum width, laterally convex. Dorsal, sub-marginal arc with ~ 22 feathered setae.

***Right mandible*** (Fig. 13c). Margin between prostheca and mola straight, smooth. Otherwise, as typical for the subgenus.

***Left mandible*** (Fig. 13d). Subtriangular process poorly developed, transparent. Margin between prostheca and mola straight, smooth. Otherwise, as typical for the subgenus.

***Hypopharynx*** (Fig. 13f). As typical for the genus.

***Maxilla*** (Fig. 13g). Maxillary palp approx. as long as galea-lacinia; palp segment II ~ 1.3× as long as segment I. Otherwise, as typical for the genus.

***Labium*** (Fig. 13e). Paraglossa dorsally with two spine-like setae near inner, distolateral margin. Labial palp with segment I 0.7× length of segments II and III combined. Segment II without distomedial protuberance, dorsally with row of four or five spine-like setae near outer, distolateral margin. Segment III slightly pentagonal, pointed, 0.8× length of segment II. Otherwise, as typical for the genus.

***Thorax*. *Sterna***. With small protuberances on sides of prosternum and close to openings of mesothoracic and metathoracic sternal apodemes (as typical for the subgenus).

***Terga*** (Figs 12c, 14c). Metanotum posteromedially with cone-like protuberance.

***Legs*** (Fig. 14a, b, i). Ratio of leg segments: fore leg 1.0:1.0:0.4:0.2, middle leg 1.0: 1.0: 0.4: 0.2, and hind leg 1.1:1.0:0.4:0.2. ***Femur***. Length ~ 3× maximum width. Surface covered with minute, pointed denticles. ***Tarsus*.** Setae along outer margin not similar as on tibia as usually, but much shorter, fine, and not ciliate. ***Claw*** with one row of six or seven denticles, and one posterior seta. Otherwise, as typical for the subgenus.

***Abdomen*. *Terga*** (Fig. 14c–e). Abdominal terga I–VIII posteromedially with cone-like, pointed protuberance, longest on terga III–V, small on VII and VIII. Posterior margin of terga: I–IX with short, rounded spines, apically carrying needle-like denticles; partly also triangular, pointed spines toward end of abdomen.

***Tergalii*** (Fig. 13h). Present on terga II–VII. Ovoid; tracheation poorly developed, pigmentation of tracheae mainly limited to main trunk; margins smooth, with short, fine, simple setae. Tergalius II as long as abdominal terga III and ½ IV combined, tergalius IV as long as terga V and ⅓ VI combined, tergalius VII reaching anterior margin of tergum IX.

***Paraproct*** (Fig. 14h). Posterior margin expanded, with denticles in distal part.

***Caudalii*** (Fig. 14f, g) Cerci without swimming setae. Paracercus with five segments.

***Pose of subimaginal gonostyli under larval cuticle*.** As typical for the subgenus.

**Subimago.** Unknown.

**Imago.** Unknown.

**Egg.** Unknown.

#### Biological aspects.

The species is only known from the upper waterfall of the Warikambon Stream at an altitude of 150 m (Fig. 29c; type locality), in the northern part of Batanta. The larvae live in fast-flowing water-films on rocks, together with the larvae of a damselfly (*Metagrion* sp.). No other *Papuanatula* species were found in the same locality.

#### Etymology.

The species name *cataracta*, meaning waterfall in Latin, refers to the habitat of the larva in the water-film on rocks in fast flowing water.

#### Distribution.

New Guinea, Batanta Island (Fig. 30).

##### ﻿Key to the species of *P. copis* species group from New Guinea (larvae)

Based on Kaltenbach et al. 2025.

**Table:** 

1	Sulawesi; patella-tibial suture absent; posterior setae on claw absent; outer margin of femur and tibia with stripe of densely situated setae	***P. normungulata***
–	New Guinea; patella-tibial suture present; posterior setae on claw present; femur and tibia with regular row of long setae	**2**
2(1)	Paracercus with 5–8 segments	**3**
–	Paracercus vestigial	**6**
3(2)	Pronotum with small, paired, triangular protuberances; fore protoptera with pair of minute protuberances at posteromedial margin	***P. webbi***
–	Pronotum and fore protoptera without protuberances	**4**
4(3)	Thoracic terga without protuberance; abdominal terga IV–VIII with small, triangular, posteromedial protuberance, oriented posteriorly (may be vestigial on II, III, and IX)	***P. parvatubera***
–	Metanotum and abdominal terga I–VIII with distinct protuberances	**5**
5(4)	Metanotum and abdominal terga I–VIII with cone-like protuberances (small on VII and VIII); anterior surface of femur with minute, pointed spines (Fig. 14i)	***P. cataracta* sp. nov.**
–	Metanotum with short, abdominal terga I–VIII with long, pointed protuberance; anterior surface of femur rough, denticulate (Fig. 11c)	***P. cukiclara* sp. nov.**
6(2)	Pronotum posteromedially with pair of protuberances	**7**
–	Pronotum without protuberance (immature larva may have minute, single, pointed protuberance at posteromedial margin)	**8**
7(6)	Abdominal terga I–VIII posteromedially with short, stout protuberances, oriented dorsally; length of mature larva 2.7–3.4 mm; tergalii narrow elongate, untracheated or poorly tracheated, margins smooth without setae; paraproct without extension, marginally without spines	***P. tuber***
–	Abdominal terga I–IX posteromedially with medium, pointed protuberances, oriented dorsoposteriorly; length of mature larva ~4.5 mm; tergalii skew ovoid, tracheation well developed, margins smooth with short, simple setae; paraproct with extension and with marginal spines	***P. pilosa***
8(6)	Fore protoptera with posteromedial minute, protuberance; metanotum and abdominal terga I–X with long, pointed protuberance	***P. batantaraja* sp. nov.**
–	Fore protoptera without protuberance; metanotum and abdominal terga I–VIII with long, pointed protuberance	**9**
9(8)	Abdominal terga I–VIII posteromedially with medium, pointed protuberances; pro-, meso- and metanotum without protuberance	***P. paratuber***
–	Abdominal terga I–VIII posteromedially with long, pointed protuberances; metanotum with conspicuous posteromedial protuberance; at least immature larva with small, acute, posteromedial protuberance on pro- and mesonotum	**10**
10(9)	Metanotum and abdominal terga I–VIII with posteromedial, long, finely pointed protuberances, oriented dorsoposteriorly; subtriangular process usually undeveloped; labial palp segment III globular	***P. copis***
–	Metanotum and abdominal terga I–VIII with posteromedial, long, pointed protuberances, hook-like bent posteriorly; subtriangular process developed; labial palp segment III oblong	***P. paracopis***

##### ﻿*Papuanatula lenos* species group (Kaltenbach et al. 2025)

**Diagnosis (larval characters).** Body dorsally without row of setae on midline; body dorsally without protuberances; femur with hypodermal macula (Figs 18a–c, 20a).

### ﻿ Papuanatula (Papuanatula) batanlenos

Taxon classificationAnimaliaEphemeropteraBaetidae

Kaltenbach, Kovács & Gattolliat sp. nov.

A8328AE5-F38E-58CE-A5CA-64B11904DDA9

https://zoobank.org/61B6A53E-7CFE-47E9-9809-E2515CFCF169

**Figure 15.**
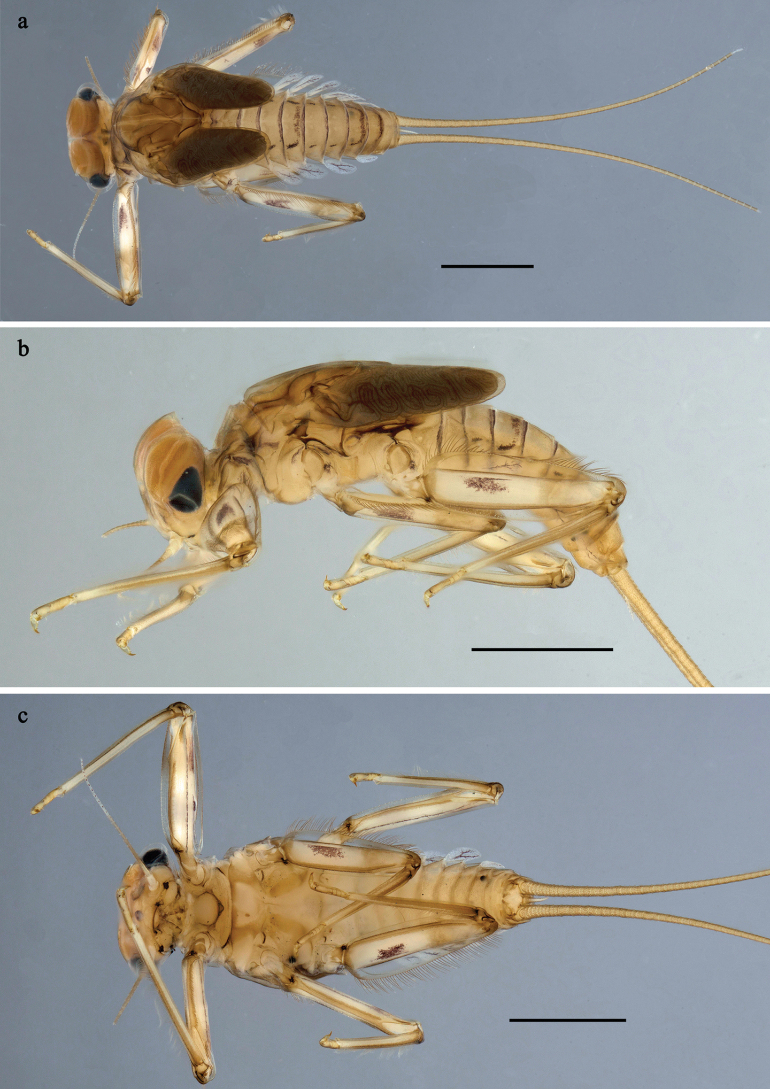
Papuanatula (Papuanatula) batanlenos sp. nov., larva, habitus. a. Dorsal view; b. Lateral view; c. Ventral view. Scale bars: 1 mm.

**Figure 16.**
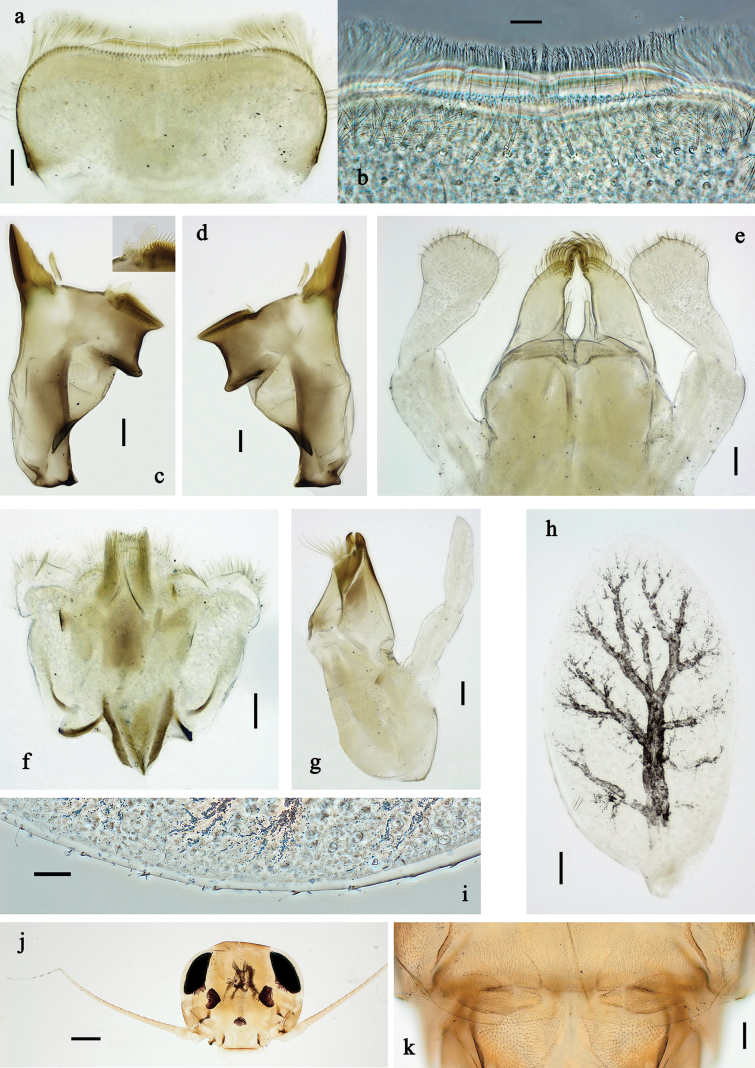
Papuanatula (Papuanatula) batanlenos sp. nov., larva. a. Labrum; b. Labrum, submarginal arc of setae; c. Left mandible; d. Right mandible; e. Labium; f. Hypopharynx and superlinguae; g. Maxilla; h, i. Tergalius IV; j. Head; k. Developing subimaginal gonostyli. Scale bars: 100 µm (j), 20 µm (a, c–h, k), 10 µm (b, i).

**Figure 17.**
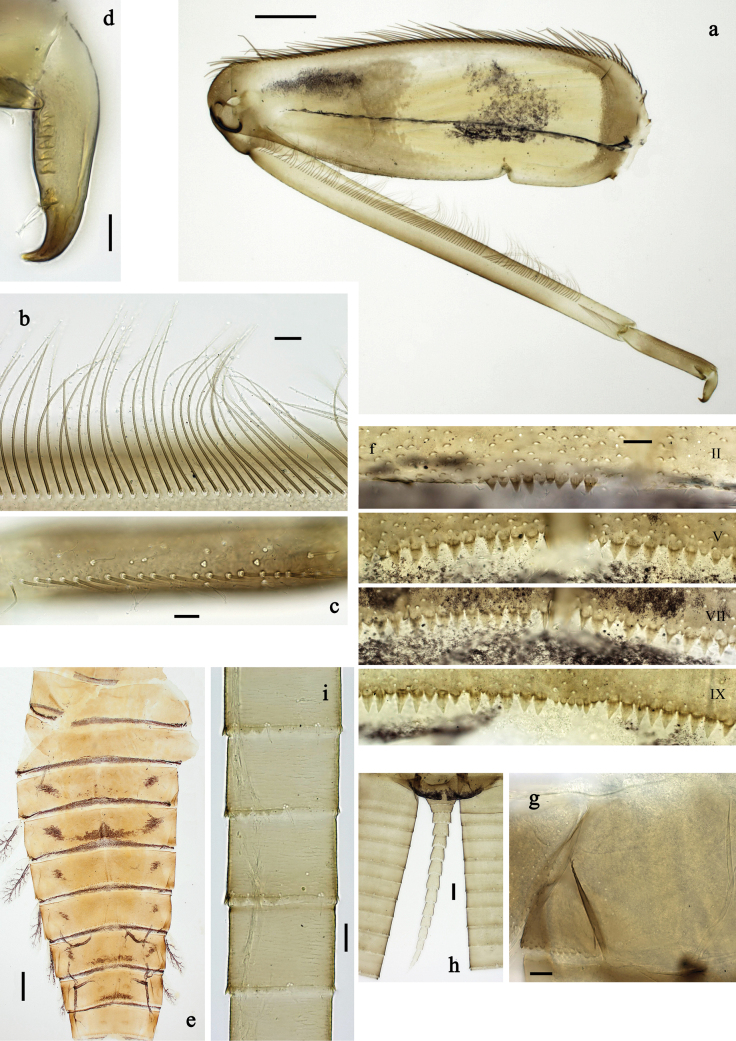
Papuanatula (Papuanatula) batanlenos sp. nov., larva. a. Middle leg; b. Middle femur, outer margin; c. Middle tarsus, outer margin; d. Middle claw; e. Abdomen; f. Abdominal terga; g. Paraproct; h. Paracercus; i. Cercus. Scale bars: 100 µm (a, e), 20 µm (h), 10 µm (b–d, f, g, i).

**Figure 18.**
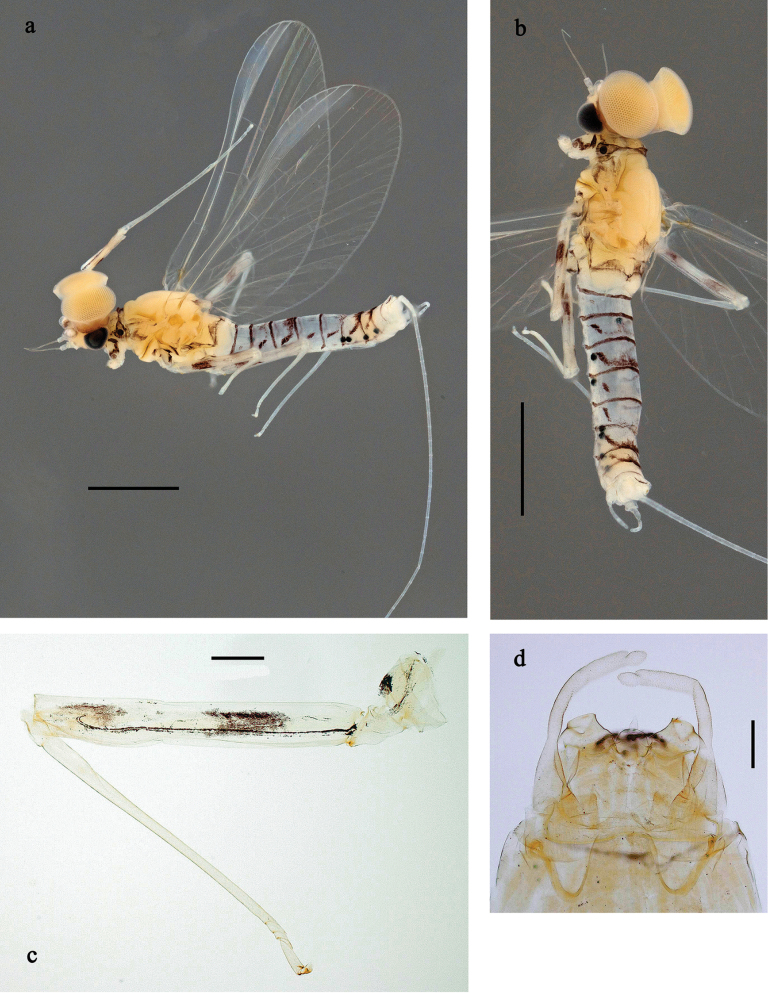
Papuanatula (Papuanatula) batanlenos sp. nov, imago, male. a. Habitus, lateral view; b. Habitus, dorsal view; c. Middle leg; d. Genitalia. Scale bars: 1 mm (a, b), 100 µm (c), 50 µm (d).

**Figure 19.**
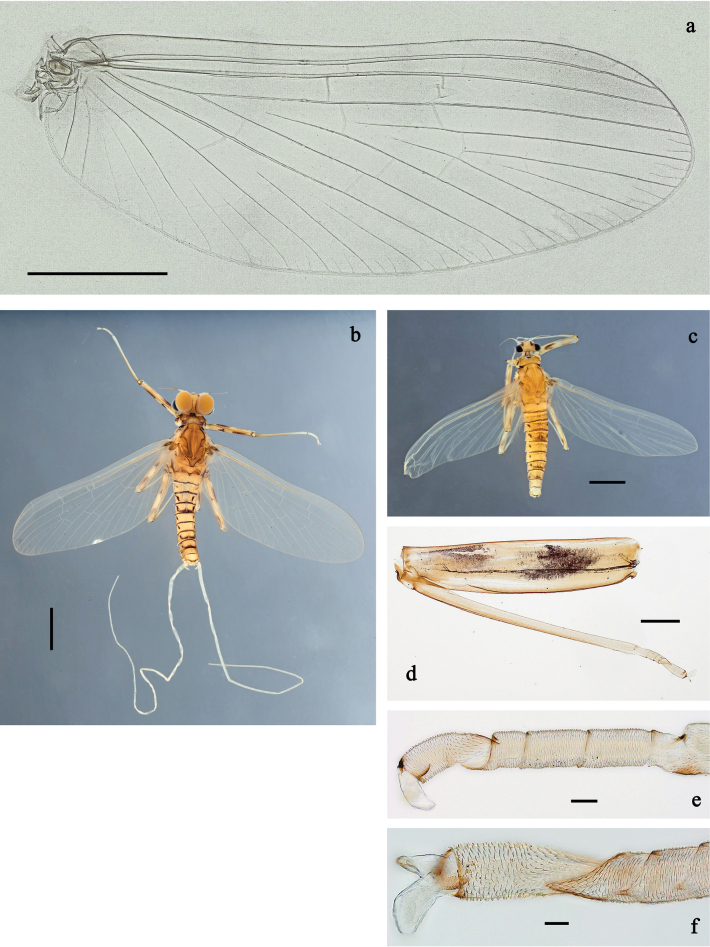
Papuanatula (Papuanatula) batanlenos sp. nov. Imago, male. a. Fore wing. Subimagos. b. Habitus, male; c. Habitus, female; d. Middle leg; e. Fore tarsus; f. Middle tarsus. Scale bars: 1 mm (a–c), 100 µm (d), 20 µm (e), 10 µm (f).

Figs 15, 16, 17, 18, 19

#### Type material.

***Holotype*.** Indonesia • larva; West Papua, Batanta Island, Kalijakut River; 00°52'27"S, 130°37'52"E; 420 m; 09.ii.2024; leg. T. Kovács; on slide; GBIFCH01221820; 2024-12, EPHTYP-11; MM. ***Paratypes*.** • 14 larvae; same data as holotype; • 2 larvae on slides; GBIFCH00975879, GBIFCH01221821 (gonostyli), GBIFCH01221824; MZL; • 12 larvae in alcohol; GBIFCH00975880, GBIFCH00975881, GBIFCH00975878, GBIFCH01581951; MZL • 3 subimagos; same data as holotype; at light, in alcohol; GBIFCH00975863 (♂, ♀), GBIFCH00975864 (♀); MZL • 2 larvae; West Papua, Batanta Island, Kalijakut River; between 00°53'39"S, 130°38'30"E and 00°53'03"S, 130°38'13"E; 52 m–182 m; 08.ii.2024; leg. T. Kovács; in alcohol; GBIFCH00975874, GBIFCH00975875; MZL • 4 larvae; West Papua, Batanta Island, valley of Waridor River; 00°51'52"S, 130°32'26"E; 55 m; 19.i.2014; leg. T. Kovács, R. Horváth, P. Juhász; 1 on slide; GBIFCH00975994; 3 in alcohol; GBIFCH00975995, GBIFCH00975996; MZL • 1 ♂ imago, 3 subimagos; West Papua, Batanta Island, valley of Kalijakut River; 00°52'49"S, 130°38'05"E; 232 m; 19.ii.2020; at light; leg. T. Kovács, R. Horváth, P. Juhász, K. Sauyai, R. Sauyai; 1 ♂ imago on slide, thorax in alcohol; GBIFCH01221780; GBIFCH00975967; 3 subimagos in alcohol; GBIFCH00975965 (♂, ♀), GBIFCH00975966 (♂); MZL • 1 imago, 2 subimagos; West Papua, Batanta Island, valley of Kalijakut River; 00°53'03"S, 130°38'13"E; 182 m; 15.ii.2023; at light; leg. T. Kovács, R. Horváth, P. Juhász, K. Sauyai, R. Sauyai; 1 ♂ imago on slide, thorax in alcohol; GBIFCH01221779, GBIFCH00975970; 1 subimago on slide; GBIFCH00975968 (♂); 1 subimago in alcohol; GBIFCH00975969 (♀); MZL • 1 ♂ imago; West Papua, Batanta Island, valley of Tanjung Lampu River; 00°53'43"S, 130°36'39"E; 175 m; 21.ii.2018; at light; leg. T. Kovács, R. Horváth, P. Juhász, K. Sauyai, R. Sauyai; on slide, thorax in alcohol; GBIFCH01221776, GBIFCH00975964; MZL • 5 subimagos; West Papua, Batanta Island, valley of Wailebet Stream; 00°53'22"S, 130°39'07"E; 150 m; 19.ii.2018; at light; leg. T. Kovács, R. Horváth, P. Juhász, K. Sauyai, R. Sauyai; 4 in alcohol; GBIFCH00975961 (1 ♂, 2 ♀), GBIFCH00975962 (♀); 1 on slide; GBIFCH00975963 (♀); MZL • 3 ♀ subimagos; West Papua, Batanta Island, valley of Wailebet Stream, waterfall; 00°53'07"S, 130°39'00"E; 285 m; 28.ii.2017; at light; leg. T. Kovács, R. Horváth, P. Juhász, K. Sauyai, R. Sauyai; in alcohol; GBIFCH00975959, GBIFCH00975960; MZL • 15 larvae; West Papua, Batanta Island, Waridor River (shallow, rocky, fast-flowing); 00°52'06"S, 130°31'30"E; 32 m; 14.ii.2025; leg. T. Kovács; 6 in alcohol; GBIFCH01582001; MZL; 9 in alcohol; 2025-12, EPHTYP-12; MM • 10 larvae; Indonesia, West Papua, Batanta Island, Kalijakut River; 00°53'03"S, 130°38'13"E; 182 m; 19.ii.2025; leg. T. Kovács; in alcohol; 2025-17, EPHTYP-13; MM • 15 larvae; West Papua, Batanta Island, Kalijakut River; 00°52'27"S, 130°37'52"E; 420 m; 20.ii.2025; leg. T. Kovács; in alcohol; 2025-18.a, EPHTYP-14; MM • 3 larvae; West Papua, Batanta Island, right side stream of Kalijakut River; 00°52'16"S, 130°37'45.5"E; 545 m; 21.ii.2025; leg. T. Kovács; in alcohol; 2025-19, EPHTYP-15; MM.

#### Diagnosis.

**Larva**. The following combination of characters distinguishes *P. batanlenos* sp. nov. from other species of *Papuanatula* s. str.: body dorsally without row of long, fine, simple setae along midline; body dorsally without protuberances; abdomen dorsally grey-brown, abdominal terga IV, VII, and VIII with trough-like, dark grey-brown marking, abdominal terga III, V, and VI with dark grey-brown, oblique, lateral streaks; femur with irregular, shoe-shaped marking in large, basal blank; paracercus with 14–16 segments; paraproct without posterior prolongation.

#### Description.

**Larva** (Figs 15–17). Body length 3.5–4.0 mm, cerci ~ 1.6× body length.

***Cuticular coloration*** (Figs 15a–c, 17e). Head, thorax and abdomen dorsally grey-brown, thorax darker; abdominal terga IV, VII, and VIII with trough-like, dark grey-brown marking; abdominal terga III, V, and VI with dark grey-brown, oblique, lateral streaks (dorsal abdominal pattern slightly varying). Head, thorax and abdomen ventrally grey-brown. Legs grey-brown, with large blank in basal area. Caudalii yellow-brown.

***Hypodermal coloration*** (Fig. 15a–c). Abdominal terga I–IX with narrow, dark brown, transverse band along posterior margin. Femur anteriorly with dark brown, irregular, shoe-shaped marking in basal ½ (somewhat variable in shape; sometimes poorly developed); posteriorly with dark brown streak distally close to outer margin.

***Head*. *Antenna*** (Fig. 16j). Length ~2.5× head length. As typical for the subgenus.

***Developing turbinate eyes in last instar male larva*** (Fig. 15a) large, round, nearly touching each other in the middle.

***Labrum*** (Fig. 16a, b). Length 0.5× maximum width, laterally convex. Dorsal, sub-marginal arc with ~ 15 feathered setae.

***Right mandible*** (Fig. 16d). Margin between prostheca and mola straight, with few minute denticles. Otherwise, as typical for subgenus.

***Left mandible*** (Fig. 16c). Margin between prostheca and mola straight, with few minute denticles. Subtriangular process with undulating margins, outer margin with denticles. Otherwise, as typical for subgenus.

***Hypopharynx*** (Fig. 16f). Medial tuft of spine-like setae laterally denser, giving the impression of a pair of tufts. Otherwise, as typical for the genus.

***Maxilla*** (Fig. 16g). Maxillary palp slightly longer than galea-lacinia; palp segment II ~ 1.2× length of segment I. Otherwise, as typical for the genus.

***Labium*** (Fig. 16e). Paraglossa with two spine-like setae on inner, distolateral margin. Labial palp with segment I ~ 0.8× length of segments II and III combined. Segment II without distomedial protuberance, dorsally with row of four spine-like setae near outer, distolateral margin. Segment III broad pentagonal, pointed, 0.8× length of segment II. Otherwise, as typical for the genus.

***Thorax*. *Sterna*** without protuberances.

***Terga*** without protuberances.

***Legs*** (Fig. 17a–d). Ratio of leg segments: fore leg 1.0:1.0:0.2:0.1, middle leg 1.0:1.0:0.2:0.1 and hind leg 1.1:1.0:0.2:0.1. ***Femur***. Length 3× maximum width. Many short, spine-like setae along ventral margin. ***Claw*** with one row of six or seven denticles, distalmost denticle with distance to other denticles; one or two posterior setae. Otherwise, as typical for subgenus.

***Abdomen*. *Terga*** (Fig. 17f). Abdominal terga without protuberances. Posterior margin of terga: I smooth, without denticles; II–IX with triangular, pointed denticles.

***Tergalii*** (Fig. 16h, i). Ovoid, tracheation rather well developed; margin with minute serration and short, fine, simple setae. Tergalius II as long as abdominal terga III, IV, and ¼ V combined, tergalius IV as long as terga V, VI and ⅓ VII combined, tergalius VII as long as terga VIII and ⅓ IX combined.

***Paraproct*** (Fig. 17g). Posterior margin without prolongation, smooth, without denticles.

***Caudalii*** (Fig. 17h, i). Cerci without swimming setae. Paracercus with 14–16 segments.

***Pose of subimaginal gonostyli under larval cuticle*** (Fig. 16k). Segment III conical. Otherwise, as typical for the subgenus.

**Subimago** (Fig. 19b–f). Body length 4.2–4.5 mm. Turbinate eyes of male ochre. Thorax brown. Wing membrane colourless, veins pale yellow-brown, base of wings brown, microtrichia pale yellow-brown. Legs very pale brown; coxa with hypodermal, dark brown fleck; femur anteriorly with hypodermal, shoe-shaped, dark brown marking in basal ½; femur posteriorly with hypodermal, dark brown distal streak and fleck in basal ½. Abdomen yellow-brown; male with hypodermal, dark brown, trough-shaped marking on terga III, IV, VII, and VIII, and sublateral, oblique, dark brown streaks on terga II, V and VI; female with same hypodermal markings, but more diffuse; all terga of both sexes with hypodermal, narrow, transvers, dark brown band on posterior margin. Cerci colourless.

***Texture*** (Fig. 19e, f). On all legs of male and female subimagos, terminal tarsomere covered with pointed microlepides; other tarsomeres covered mostly with blunt microlepides, with pointed microlepides near apex.

**Imago, male** (Figs 18a–d, 19a). Body length ~ 3.8 mm. Head and antennae pale brown. Turbinate eyes pale brown, wide, with facetted surfaces round. Thorax pale brown. Fore wing with membrane colourless, base of wings brownish. Pterostigma with three or four oblique crossveins, basal two crossveins nearly complete. Legs light brownish; coxa with hypodermal, dark brown fleck; femur anteriorly with hypodermal, shoe-shaped, dark brown marking in basal ½; femur posteriorly with hypodermal, dark brown distal streak and fleck in basal ½. Abdominal segments I and VII–X pale brown, II–VI transparent, giving a blueish impression; with hypodermal, dark brown, trough-shaped marking on terga III, IV, VII, and VIII, and sublateral, oblique, dark brown streaks on terga II, V, and VI. Cerci colourless.

***Genitalia*** (Fig. 18d). Unistyliger slightly widened apically, with lateral margin slightly concave. Gonostylus with segment I conical and separated from segment II by concavity. Segment II slightly wider in distal ½, bent inward. Terminal segment III narrower than segment II, ovoid, length ~ 1.5× maximal width. Penial bridge with poorly expressed membranous projection between unistyligers.

**Imago, female**. Unknown.

**Egg.** Unknown. Eggs extracted from female subimagos are undeveloped.

#### Biological aspects.

It is a common species in most riverine habitat types in Batanta, living in the northern and southern watercourses of the island: Kalijakut (Fig. 29a, d (type locality), f), Tanjung Lampu and Waridor (Fig. 29e) Rivers, and Wailebet Stream. It is found from small, high-falling mountain tributaries to the lower, flat sections of the largest rivers at altitudes between 32 m and 545 m. Apart from the extreme habitat of *P. cataracta* sp. nov., it co-occurs with all *Papuanatula* species living in Batanta: *P. batantaraja* sp. nov., *P. cukiclara* sp. nov., *P. horvathrobi* sp. nov., and *P. longabranchias* sp. nov.

#### Etymology.

The species name *batanlenos* refers to the type locality on Batanta island and its belonging to the *lenos* species group.

#### Distribution.

New Guinea, Batanta Island (Fig. 30).

##### ﻿Key to the species of *P. lenos* species group from New Guinea (larvae)

Based on Kaltenbach et al. 2025.

**Table:** 

1	New Britain; femur anteriorly with large, brown, triangular macula	***P. vaisisi***
–	New Guinea; femur anteriorly with macula other than triangular	**2**
2(1)	Femur anteriorly with dark brown, shoe-shaped macula inside large, proximal blank; hypopharynx apically with “paired” tufts of seta-like spines	**5**
–	Femur anteriorly with macula other than shoe-shaped in proximal ½; hypopharynx with usual, unpaired tuft of seta-like spines	**3**
3(2)	Femur anteriorly with brown, hypodermal streak in proximal ½; posterior margin of abdominal terga with heterogenous, sharply pointed denticles	***P. zebrata***
–	Femur anteriorly with red-brown to dark brown or blackish, oblong to drop-shaped hypodermal marking in mediodistal area; posterior margin of abdominal terga with regular, triangular denticles	**4**
4(3)	Thorax dorsally without distinct markings; femur with row of short, spine-like setae on inner margin; tergalii margin smooth, with short, fine, simple setae	***P. lenos***
–	Pronotum with large, dark brown marking medially on anterior margin, narrow dark brown band along posterior margin of pronotum and anterior margin of mesonotum; femur with many medium, spine-like setae along inner margin; tergalii margin with minute serration and short, fine, simple setae	***P. paralenos***
5(2)	Abdomen with dark grey-brown, trough-like markings on terga IV, VII, and VIII, laterally with oblique streaks on terga III, V and VI; paracercus with 14–16 segments	***P. batanlenos* sp. nov.**
–	Abdomen with dark brown, trough-like marking on tergum IV, laterally without distinct oblique streaks; paracercus with 10–12 segments	***P. heterochaeta***

### ﻿Subgenus Papuafiliola

Taxon classificationAnimaliaEphemeropteraBaetidae

Kaltenbach, Kluge & Gattolliat, 2025

DDF16181-DA04-5D39-AAEB-357F74225338

Figs 20, 21, 22, 23, 24, 25, 26, 27, 28

#### Diagnosis

**(larval characters; see Kaltenbach et al. 2025: 318).** Antennal flagellum distally without brown dots; labrum widest at base, dorsally with few submarginal, simple setae (Fig. 24b); both mandibles without prolonged, blade-like incisor (Fig. 24c, d); labial palp with distomedian projection on segment II (Fig. 24e); outer side of femur with regular row of long, slender, flattened, parallel-sided setae with blunt apex (Fig. 25a, b); tibia with regular row of similar setae (Fig. 25a, c); tarsus on inner margin with distalmost seta not longer or only slightly longer than other setae.

### ﻿ Papuanatula (Papuafiliola) horvathrobi

Taxon classificationAnimaliaEphemeropteraBaetidae

Kovács, Kaltenbach & Gattolliat sp. nov.

A884DA10-6BD1-50DA-ABC5-47E8FAF2D79D

https://zoobank.org/CBB57F98-CF73-43AE-AD47-58D6D1D8F031

Figs 20, 21, 22

#### Type material.

***Holotype*.** Indonesia • larva; West Papua, Batanta Island, Waibin River; between 00°49'21"S, 130°45'57"E and 00°50'02"S, 130°45'25"E; 20 m–45 m; 05.ii.2024; leg. T. Kovács; on slide; GBIFCH00975876; 2024-9, EPHTYP-16; MM. ***Paratypes*.** 1 larva; same data as holotype; on slide; GBIFCH00975913; MZL • 1 larva; West Papua, Batanta Island, Warai Stream; between 00°50'51"S, 130°35'14"E and 00°51'12"S, 130°35'20"E; 225 m–315 m; 21.ii.2024; leg. T. Kovács; on slide; GBIFCH00975877; MZL • West Papua, Batanta Island, Waridor River (shallow, rocky, fast-flowing); 00°52'06"S, 130°31'30"E; 32 m; 14.ii.2025; leg. T. Kovács; 4 in alcohol; GBIFCH01582003; MZL; 12 in alcohol; 2025-12, EPHTYP-17; MM.

#### Diagnosis.

**Larva.** The following combination of characters distinguishes *P. horvathrobi* sp. nov. from other species of Papuanatula (Papuafiliola): thorax and abdomen dorsally without protuberances; femur with large blank in posterior ⅔; femur and abdomen without brown hypodermal maculae; tergalii oblong; paracercus with 15 segments.

#### Description.

**Figure 20.**
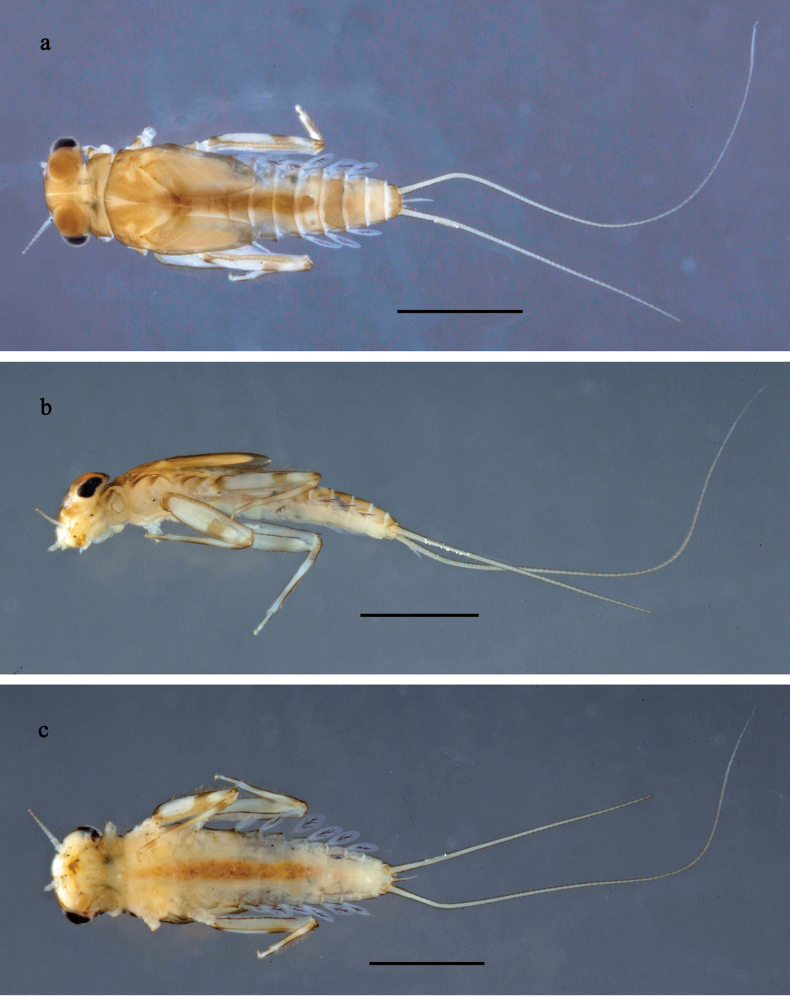
Papuanatula (Papuafiliola) horvathrobi sp. nov., larva, habitus. a. Dorsal view; b. Lateral view; c. Ventral view. Scale bars: 1 mm.

**Figure 21.**
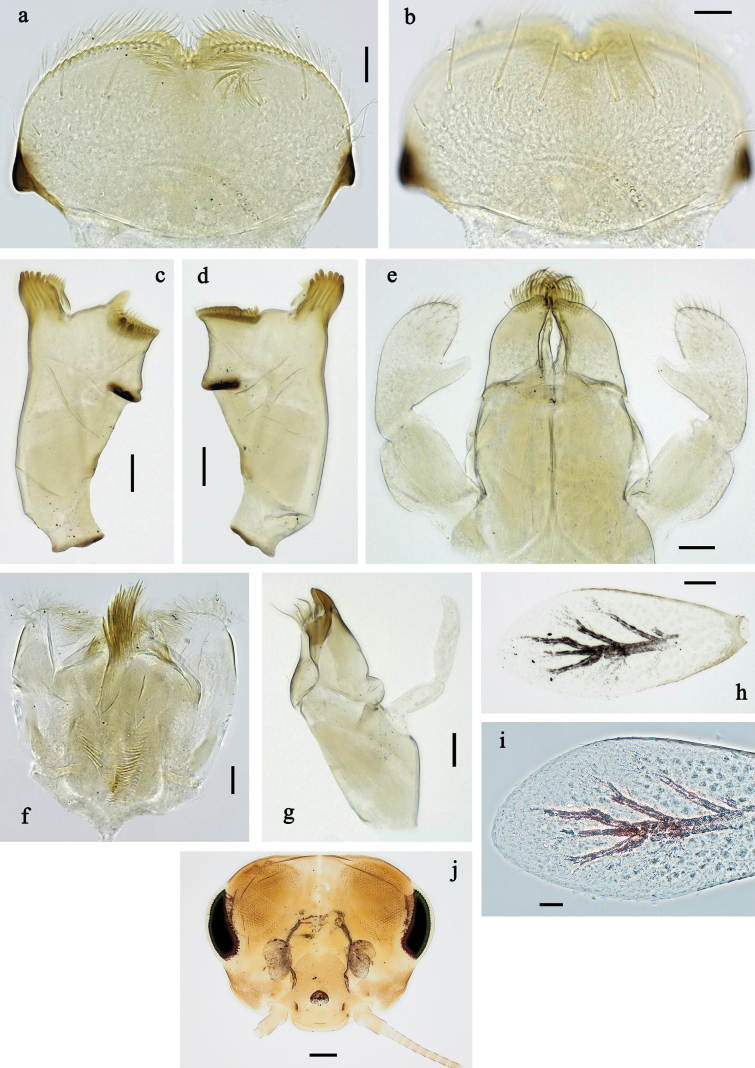
Papuanatula (Papuafiliola) horvathrobi sp. nov., larva. a. Labrum; b. Labrum, submarginal arc of setae; c. Left mandible; d. Right mandible; e. Labium; f. Hypopharynx and superlinguae; g. Maxilla; h, i. Tergalius V; j. Head. Scale bars: 50 µm (j), 20 µm (c–h), 10 µm (a, b, i).

**Figure 22.**
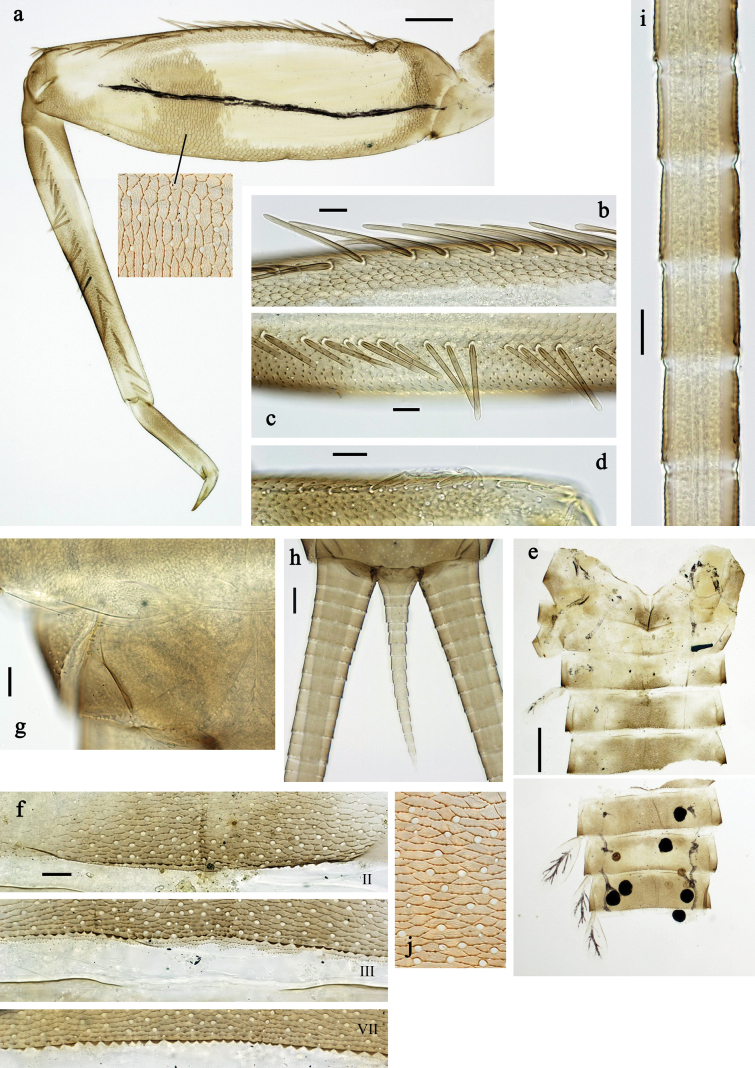
Papuanatula (Papuafiliola) horvathrobi sp. nov., larva. a. Hind leg; b. Hind femur, outer margin; c. Hind tibia, anterior surface; d. Hind tarsus, outer margin; e. Abdomen; f. Abdominal terga; g. Paraproct and developing subimaginal gonostylus; h. Paracercus; i. Cercus; j. Abdominal tergum IV, surface. Scale bars: 100 µm (e), 50 µm (a), 20 µm (h), 10 µm (b–d, f, g, i).

***Larva*** (Fig. 20–22). Body length ~ 3.0 mm, cerci ~ 1.4× body length.

***Cuticular coloration*** (Figs 20a, b, 22a). Head, thorax, and abdomen dorsally brown. Head, thorax and abdomen ventrally beige. Legs brown, femur with large blank in posterior ⅔, and distally with small blank area. Caudalii light brown.

***Hypodermal coloration*** (Fig. 20a, b). Head, thorax, abdomen, and legs without hypodermal markings.

***Head*. *Antenna*.** Length ~ 2.5× head length. Otherwise, as typical for the subgenus.

***Developing turbinate eyes in last instar male larva*** (Figs 20a, 21j) large, roundish, with some distance to each other.

***Labrum*** (Fig. 21a, b). Length 0.6× maximal width. With reduced, submarginal arc of three long, simple setae. Otherwise, as typical for subgenus.

***Right mandible*** (Fig. 21d). Incisor with five denticles; kinetodontium with three denticles, inner lateral margin with row of small denticles, becoming smaller and finer toward base, and with row of short, fine setae; margin between prostheca and mola slightly convex, with few minute denticles. Otherwise, as typical for subgenus.

***Left mandible*** (Fig. 21c). Incisor with five denticles, kinetodontium with three denticles; margin between prostheca and subtriangular process straight, with minute denticles. Otherwise, as typical for subgenus.

***Hypopharynx*** (Fig. 21f) with well-developed tuft of long, spine-like setae. Otherwise, as typical for the genus.

***Maxilla*** (Fig. 21g). Maxillary palp slightly longer than galea-lacinia; segment I 1.4× length of segment II. Otherwise, as typical for the genus.

***Labium*** (Fig. 21e) Paraglossa dorsally with three spine-like setae near inner, distolateral margin. Labial palp with segment I 0.7× length of segments II and III combined. Segment II with narrow thumb-like distomedial protuberance, slightly bent distad, dorsally with one spine-like seta near outer, distolateral margin. Segment III oblique conical, apically rounded, approx. as long as segment II. Otherwise, as typical for the genus.

***Thorax*. *Sterna*** without protuberances.

***Terga*.** Without long setae on midline; without protuberance.

***Legs*** (Fig. 22a–d). Ratio of leg segments: fore leg 1.1:1.0:0.3:0.1, middle leg 1.2:1.0:0.3:0.1 and hind leg 1.5:1.0:0.4:0.2. ***Femur***. Length ~ 3× maximum width. Surface of brown areas with minute denticles. ***Tarsus*.** With regular row of medium, apically rounded setae along outer margin. ***Claw*** with one row of 11 denticles; one posterior seta. Otherwise, as typical for subgenus.

***Abdomen*. *Terga*** (Fig. 22e, f, j). Abdominal terga without long setae on midline; abdominal terga without median or submedian elevations or protuberances; surface with minute denticles. Posterior margins of abdominal terga: I smooth, without denticles, II–IX with very small, triangular denticles.

***Tergalii*** (Fig. 21h, i). Present on terga II–VII, oblong; tracheation well-developed, pigmentation not reaching margins. Each tergalius with anal rib longer than costal rib; ribs with minute, irregularly situated denticles on dorsal side. Tergalius II as long as terga III and ⅔ IV combined, tergalius IV as long as terga V and ⅔ VI combined, tergalius VII as long as terga VIII and ⅔ IX combined.

***Paraproct*** (Fig. 22g) without posterior prolongation. Posterior margin with small denticles.

***Caudalii*** (Fig. 22h, i). Cerci without swimming setae. Paracercus with 15 segments.

***Pose of subimaginal gonostyli under larval cuticle*** unknown.

**Subimago.** Unknown.

**Imago.** Unknown.

**Egg**. Unknown.

#### Biological aspects.

The species is known from three watercourses in the northern part of Batanta (Waibin River (type locality), Waridor River, and Warai Stream), at altitudes from 20–315 m. The largest numbers were collected at a ford of the Waridor River at an altitude of 32 m (Fig. 29e), where *P. batanlenos* sp. nov., was also present in large numbers.

#### Etymology.

The new species is dedicated to Róbert Horváth, a Hungarian ornithologist, who initiated and organized the Hungarian research program on Biodiversity in Batanta in 2010. Since then, he gave continuous support in various aspects of the ten research trips conducted to date.

#### Distribution.

New Guinea, Batanta Island (Fig. 30).

### ﻿ Papuanatula (Papuafiliola) longabranchias

Taxon classificationAnimaliaEphemeropteraBaetidae

Kaltenbach, Kovács & Gattolliat sp. nov.

993AB681-5BE7-5EF5-92FC-ECA16C6A659C

https://zoobank.org/0AB4B115-0A8E-4B40-AF33-6997824F06C5

**Figure 23.**
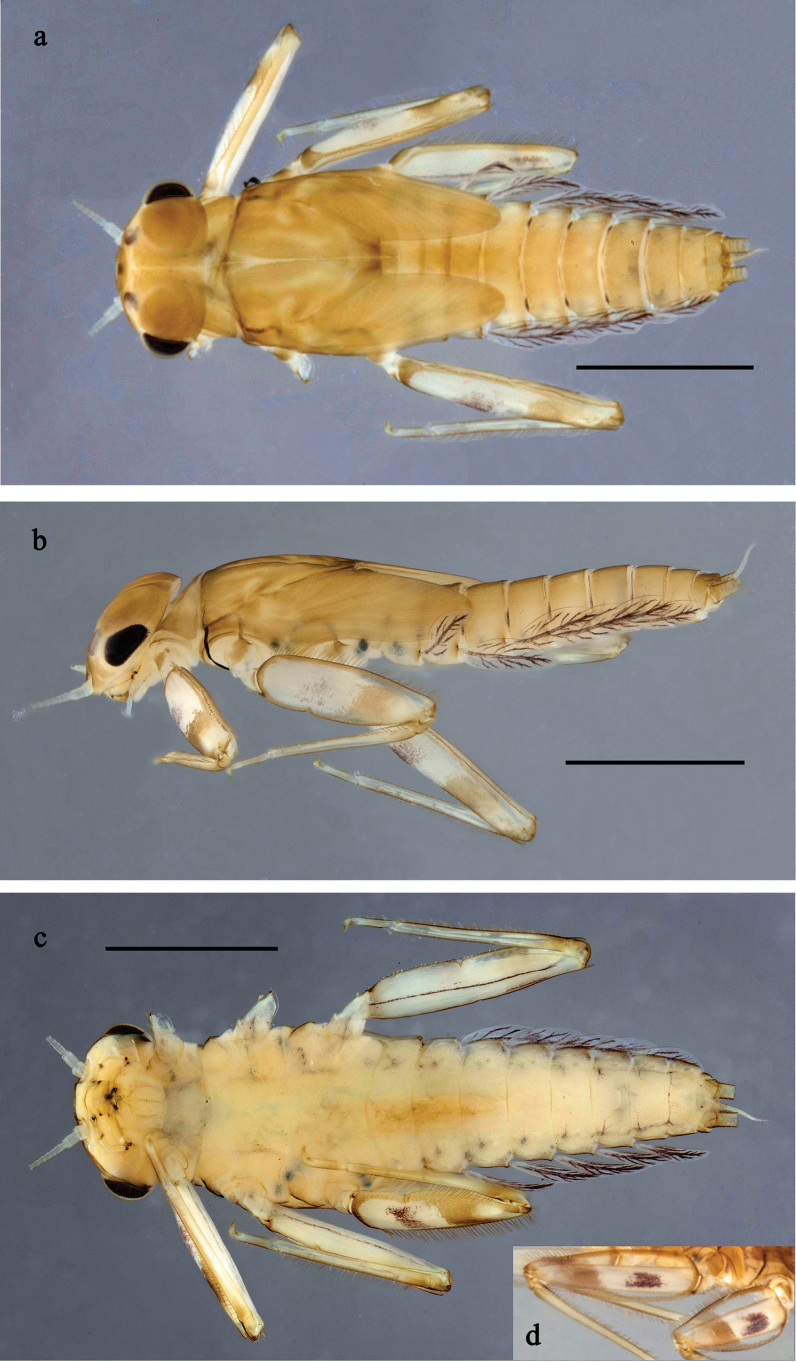
Papuanatula (Papuafiliola) longabranchias sp. nov., larva, habitus. a. Dorsal view; b. lateral view; c. ventral view; d. legs. Scale bars: 1 mm.

**Figure 24.**
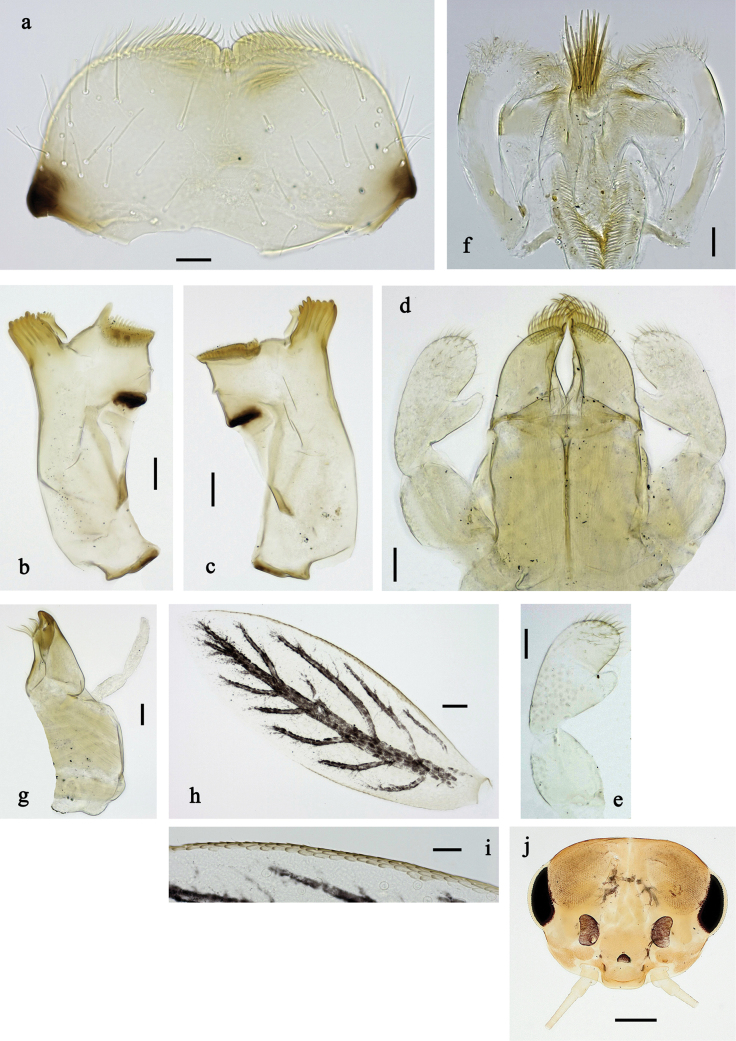
Papuanatula (Papuafiliola) longabranchias sp. nov., larva. a. Labrum; b. Left mandible; c. Right mandible; d. Labium; e. Labial palp; f. Hypopharynx and superlinguae; g. Maxilla; h, i. Tergalius III; j. Head. Scale bars: 100 µm (j), 20 µm (b–d, f–h), 10 µm (a, e, i).

**Figure 25.**
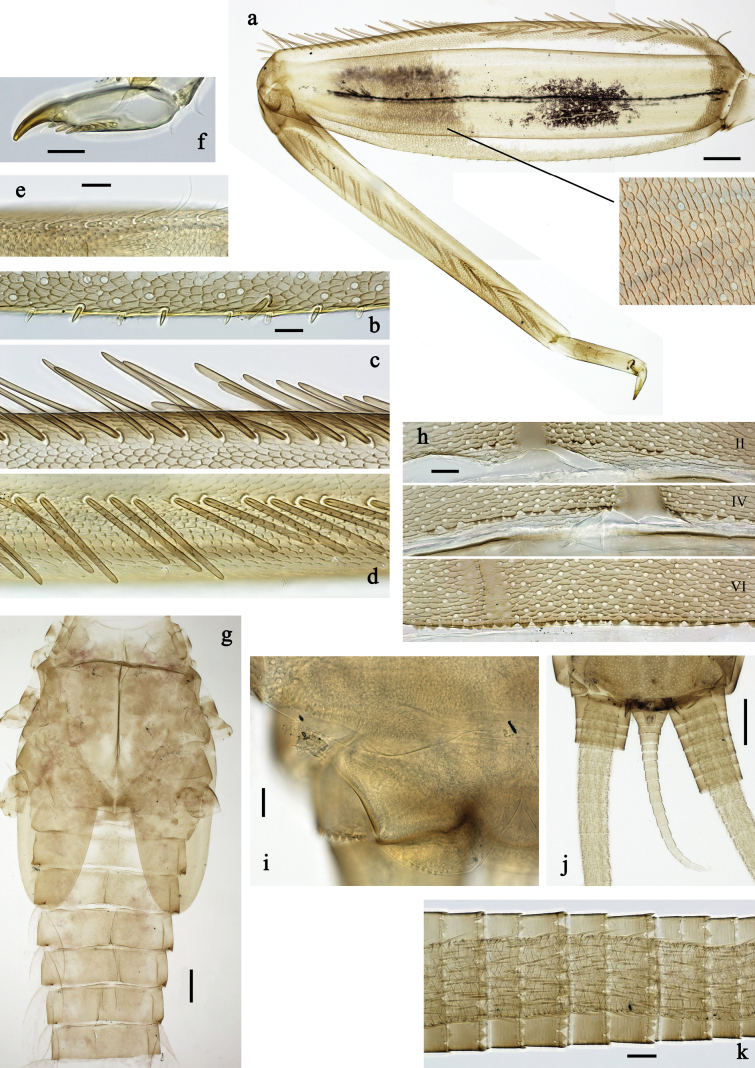
Papuanatula (Papuafiliola) longabranchias sp. nov., larva. a. Hind leg; b. Hind femur, inner margin; c. Hind femur, outer margin; d. Hind tibia, anterior surface; e. Hind tarsus, outer margin; f. Hind claw; g. Thorax, abdomen; h. Abdominal terga; i. Paraproct; j. Paracercus; k. Cercus. Scale bars: 100 µm (g), 50 µm (a), 20 µm (j), 10 µm (b–f, h, I, k).

**Figure 26.**
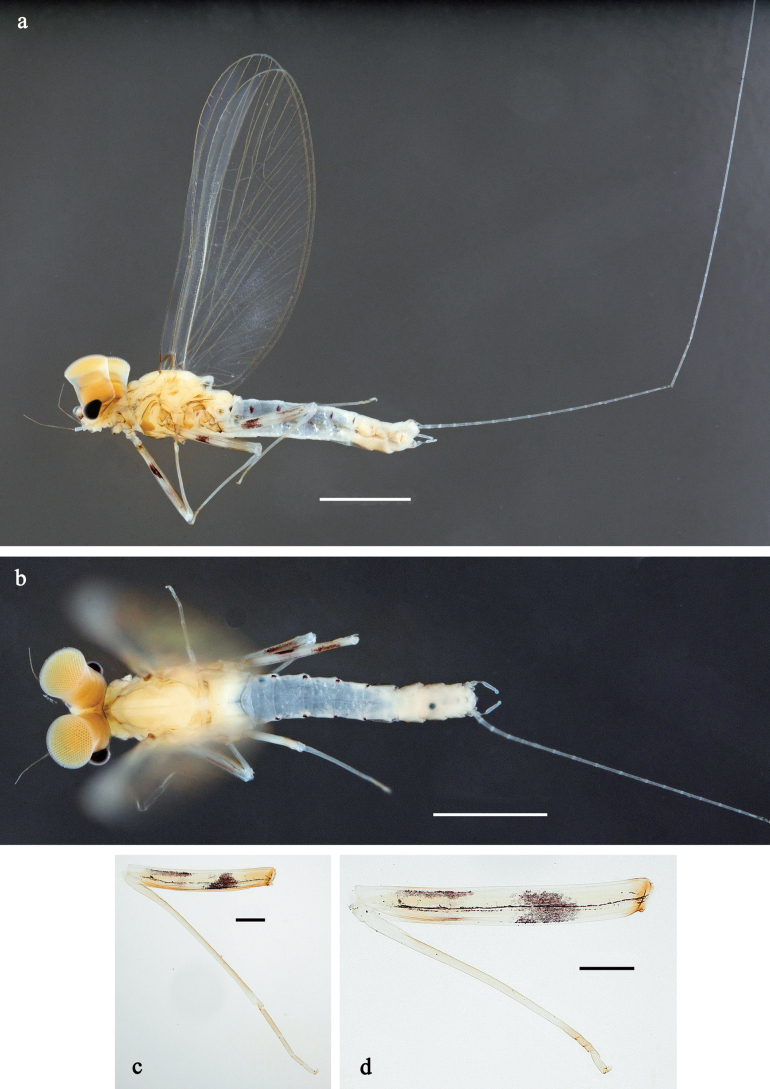
Papuanatula (Papuafiliola) longabranchias sp. nov, imago, male. a. Habitus, lateral view; b. Habitus, dorsal view; c. Fore leg; d. Hind leg. Scale bars: 1 mm (a, b), 100 µm (c, d).

**Figure 27.**
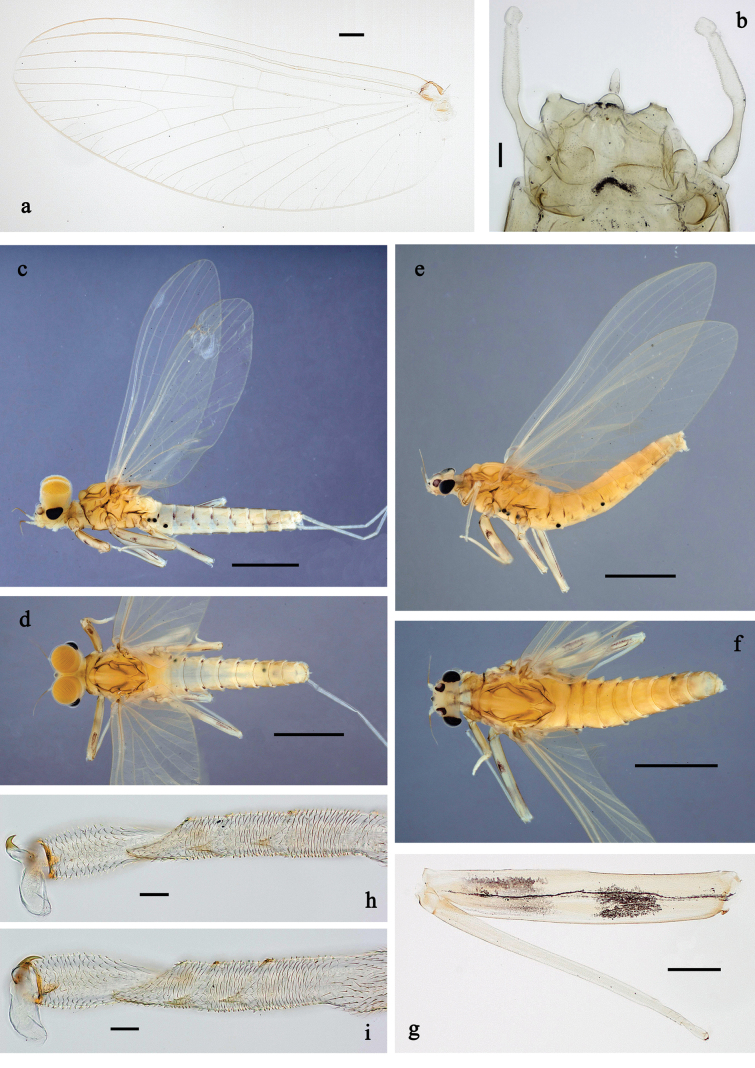
Papuanatula (Papuafiliola) longabranchias sp. nov. Imago, male. a. Fore wing; b. Genitalia. Subimagos. c, d. Habitus, male (lateral view, dorsal view); e, f. Habitus, female (lateral view, dorsal view); g. Hind leg; h. Fore tarsus; i. Hind tarsus. Scale bars: 1 mm (c–f), 100 µm (a, g), 20 µm (b, h), 10 µm (i).

Figs 23, 24, 25, 26, 27

#### Type material.

***Holotype*.** Indonesia • larva; West Papua, Batanta Island, Kalijakut River; 00°52'27"S, 130°37'52"E; 420 m; 09.ii.2024; leg. T. Kovács; on slide; GBIFCH00975882; 2024-12, EPHTYP-18; MM. ***Paratypes*.** 2 larvae; same data as holotype; on slides; GBIFCH00975883, GBIFCH00975884; MZL • 3 ♂ imagos, 30 subimagos; West Papua, Batanta Island, valley of Kalijakut River; 00°53'03"S, 130°38'13"E; 182 m; 15.ii.2023; at light; leg. T. Kovács, R. Horváth, P. Juhász, K. Sauyai, R. Sauyai; 1 imago on slide, thorax in alcohol; GBIFCH01221781, GBIFCH00975811; 2 imagos in alcohol; GBIFCH00975939, GBIFCH00975940; 30 subimagos; 28 in alcohol; GBIFCH00975941 (13♂, 10♀), GBIFCH00975942 (♂), GBIFCH00975943 (♂), GBIFCH00975944 (2♂, 1♀); 2 on slides; GBIFCH01221833 (♀), GBIFCH01221834 (♂); MZL • 15 subimagos; West Papua, Batanta Island, valley of Tanjung Lampu River; 00°53'43"S, 130°36'39"E; 18.ii.2020; at light; leg. T. Kovács, R. Horváth, P. Juhász; in alcohol; GBIFCH00975952 (9♂, 4♀), GBIFCH00975953 (♂), GBIFCH00975954 (♂); MZL • 15 subimagos; West Papua, Batanta Island, valley of Kalijakut River; 00°52'49"S, 130°38'05"E; 232 m; 19.ii.2020; at light; leg. T. Kovács, R. Horváth, P. Juhász, K. Sauyai, R. Sauyai; in alcohol; GBIFCH00975948 (6♂, 6♀), GBIFCH00975949 (♂), GBIFCH00975950 (♂), GBIFCH00975951 (♂); MZL • 7 subimagos (4♂, 3♀); West Papua, Batanta Island, valley of Tanjung Lampu River; 00°53'43"S, 130°36'39"E; 175 m; 21.ii.2018; at light; leg. T. Kovács, R. Horváth, P. Juhász, K. Sauyai, R. Sauyai; in alcohol; GBIFCH00975945, GBIFCH00975946, GBIFCH00975947; MZL • 3 larvae; West Papua, Batanta Island, Kalijakut River; 00°52'27"S, 130°37'52"E; 420 m; 20.ii.2025; leg. T. Kovács; 2 in alcohol; GBIFCH01582002; MZL; 1 in alcohol; 2025-18.a, EPHTYP-19; MM • 6 larvae; West Papua, Batanta Island, Kalijakut River; 00°53'03"S, 130°38'13"E; 182 m; 19.ii.2025; leg. T. Kovács; in alcohol; 2025-17, EPHTYP-20; MM.

#### Diagnosis.

**Larva.** The following combination of characters distinguishes *P. longabranchias* sp. nov. from other species of Papuanatula (Papuafiliola): abdomen dorsally without long setae on midline; without median protuberances on abdominal terga; abdomen dorsally pale brown, with short, dark brown, lateral streaks along anterior margin of terga I–VII (VIII); femur with dark brown, shoe-shaped marking (marking longer than broad); tergalii very long and narrow; paracercus with 17 or 18 segments. The larva is hardly distinguished from *P. stenophylla* Kaltenbach, Kluge & Gattolliat, 2025.

#### Description.

***Larva*** (Fig. 23–25). Body length 3.1–3.7 mm, cerci broken.

***Cuticular coloration*** (Figs 23a–c, 25g). Head, thorax and abdomen dorsally pale brown, mesonotum with some lighter areas, metanotum medially darker brownish. Legs pale brown, with large blank in basal part and small blank area in distal part. Head, thorax and abdomen ventrally beige. Cerci light brownish.

***Hypodermal coloration*** (Fig. 23a, b). Abdomen dorsally with short, dark brown, lateral streaks along anterior margin of terga I–VII (VIII). Anterior side of femur with shoe-shaped, dark brown marking in blank area, longer than wide. Posterior side of femur with dark brown streak in outer distal area.

***Head*. *Antenna*** (Fig. 24j). As typical for the subgenus, with each flagellomere symmetric, cylindrical.

***Developing turbinate eyes in last instar male larva*** (Figs 23a, 24j) large, roundish.

***Labrum*** (Fig. 24a) Length 0.6× maximal width. With reduced, submarginal arc of three long, simple setae. Otherwise, as typical for subgenus.

***Right mandible*** (Fig. 24c) Incisor with five denticles; kinetodontium with three denticles, inner lateral margin with row of small denticles, becoming smaller and finer toward base, and with row of short, fine setae; margin between prostheca and mola slightly convex, smooth. Otherwise, as typical for subgenus.

***Left mandible*** (Fig. 24b) Incisor with five denticles, kinetodontium with four denticles; margin between prostheca and subtriangular process straight, with few minute denticles. Otherwise, as typical for subgenus.

***Hypopharynx*** (Fig. 24f) with tuft of long, straight, stout, spine-like setae. Otherwise, as typical for the genus.

***Maxilla*** (Fig. 24g). Maxillary palp approx. as long as galea-lacinia; segment II ~ 1.5× length of segment I. Otherwise, as typical for the genus.

***Labium*** (Fig. 24d, e) Paraglossa dorsally with three spine-like setae near inner, distolateral margin. Labial palp with segment I 0.8× length of segments II and III combined. Segment II with narrow thumb-like distomedial protuberance, slightly bent distad (protuberance somewhat varying), dorsally with one spine-like seta near outer, distolateral margin. Segment III oblong, apically rounded, ~ 0.9× length of segment II. Otherwise, as typical for the genus.

***Thorax*. *Sterna*** without protuberances.

***Terga*.** Without long setae on midline; without protuberances.

***Legs*** (Fig. 25a–f). Ratio of leg segments: fore leg 1.1:1.0:0.3:0.1, middle leg 1.2:1.0:0.3:0.1 and hind leg 1.2:1.0:0.3:0.2. ***Femur*.** Length ~ 3.6× maximum width. Surface rough in brown areas. ***Tarsus*.** With regular row of medium, apically rounded setae along outer margin, similar as on tibia, but much shorter. ***Claw*** with one row of 9–11 denticles; one posterior seta. Otherwise, as typical for subgenus.

***Abdomen*. *Terga*** (Fig. 25g, h). Abdominal terga without long, fine setae on midline, without median or submedian elevations or protuberances. Surface of abdominal terga rough, with numerous short sensillae, without scattered fine simple setae. Posterior margins of abdominal terga: I smooth, without denticles, II–IX with very small, triangular, apically rounded, dark brown denticles.

***Tergalii*** (Fig. 24h, i). Present on abdominal terga II–VII; long and narrow. Tracheae and pigmentation well-developed, reaching margins; ribs with very small, irregularly situated denticles on dorsal side. Tergalius II as long as terga III and IV combined; tergalius IV as long as terga V, VI, and ½ VII combined; tergalius VII as long as terga VIII, IX, and ½ X combined.

***Paraproct*** (Fig. 25i). Without posterior prolongation; with small spines on posterior margin.

***Caudalii*** (Fig. 25j, k). Cerci without swimming setae. Paracercus with 17 or 18 segments.

***Pose of subimaginal gonostyli under larval cuticle***. Unknown.

**Subimago** (Fig. 27c–i). Body length 3.6–4.1 mm. Turbinate eyes of male ochre. Thorax pale brown. Wing membrane colourless, veins pale brown, microtrichia pale brown. Legs very pale brown; femur anteriorly with hypodermal, shoe-shaped, dark brown marking in basal ½; femur posteriorly with hypodermal, dark brown distal streak. Abdomen of female pale brown; male with abdominal segments I and VII–X pale brown, II–VI nearly colourless; both sexes with hypodermal, sublateral, transvers, dark brown short streaks on posterior margin of terga I–VII. Cerci colourless.

***Texture*** (Fig. 27h, i). On all legs of male and female subimagos, terminal tarsomere covered with pointed microlepides only; other tarsomeres covered mostly with blunt microlepides, with pointed microlepides near apex.

**Imago, male** (Figs 26a–d, 27a, b). Body length ~ 3.8 mm. Head pale brown, antennae pale brown, turbinate eyes pale brown, wide, with facetted surfaces round. Thorax pale brown to brown. Fore wing with membrane colourless, veins pale brown, base of RA and costal brace brownish. Pterostigma with two or three oblique crossveins, basalmost crossvein nearly complete, others incomplete. Legs brownish; femur with hypodermal, dark brown, shoe-shaped macula in basal ½ of anterior side; femur on posterior side distally with hypodermal, dark brown streak. Abdominal segments I and VII–X pale brown, II–VI nearly colourless; with hypodermal, dark brown, short, sublateral, transvers streaks on posterior margin of terga I–VII. Cerci colourless.

***Genitalia*** (Fig. 27b). Unistyliger slightly widened apically, with median and lateral margins slightly convex. Gonostylus with segment I on lateral and median side convex and separated from segment II by concavity. Segment II almost equally wide and straight all over its length. Terminal segment III slightly wider than segment II, cube-like. Penial bridge with poorly expressed membranous projection between unistyligers.

**Imago, female.** Unknown.

**Figure 28.**
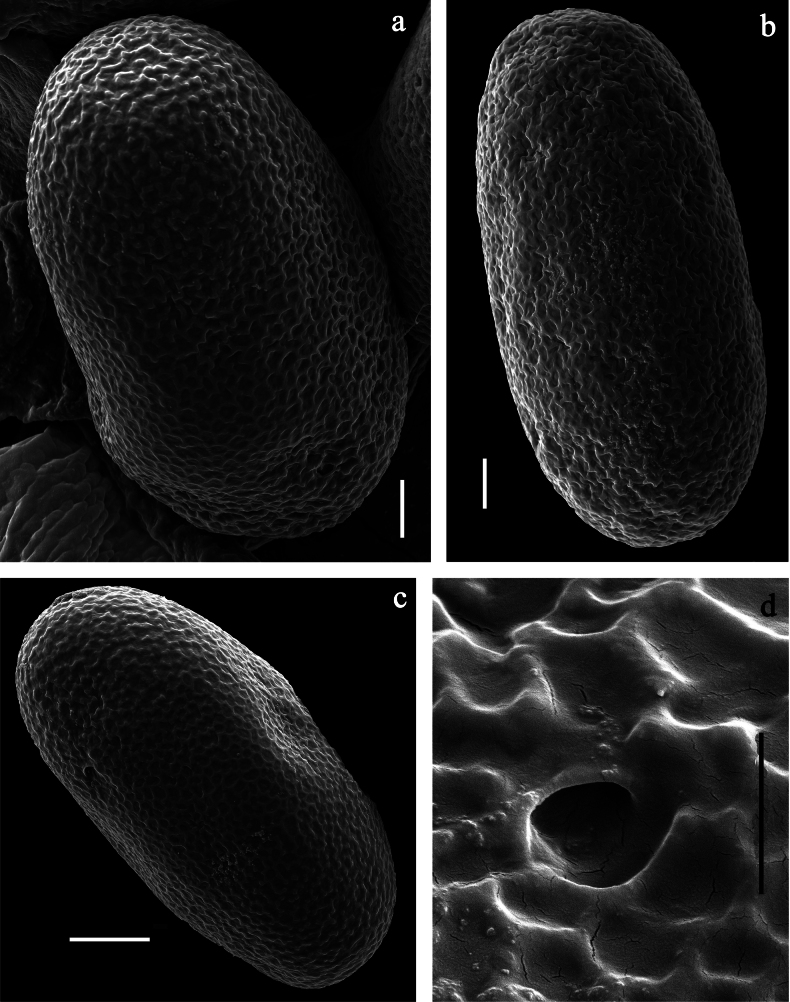
Papuanatula (Papuafiliola) longabranchias sp. nov. a–c. Eggs; d. Micropyle. Scale bars: 10 µm (a–c), 5 µm (d).

**Egg** (Fig. 28a–d). Elongate ovoid. Chorion entirely covered with ridges forming a net-like relief.

#### Comparison.

The larva of Papuanatula (Papuafiliola) longabranchias sp. nov. is hardly different from Papuanatula (Papuafiliola) stenophylla Kaltenbach, Kluge & Gattolliat, 2025: *P. longabranchias* sp. nov. with hypodermal marking on anterior side of femur shoe-shaped, usually longer than wide (Fig. 23d); no short, fine, simple setae on surface of abdominal tergites (Fig. 25h); paracercus with 17 or 18 segments (Fig. 25j). *P. stenophylla* with hypodermal marking on anterior side of femur usually like a band, wider than long; scattered short, fine, simple setae on surface of abdominal terga; paracercus with ~ 15 segments (Kaltenbach et al. 2025: figs 132a, b, 135d). However, male imagos are clearly different: *P. longabranchias* sp. nov. has pale brown turbinate eyes (Fig. 26a, b), whereas *P. stenophylla* has dull-red turbinate eyes (Kaltenbach et al. 2025: fig. 137a, d). The eggs of both species are identical (Fig. 28a–d; Kaltenbach et al. 2025: fig. 138a).

#### Biological aspects.

**Figure 29.**
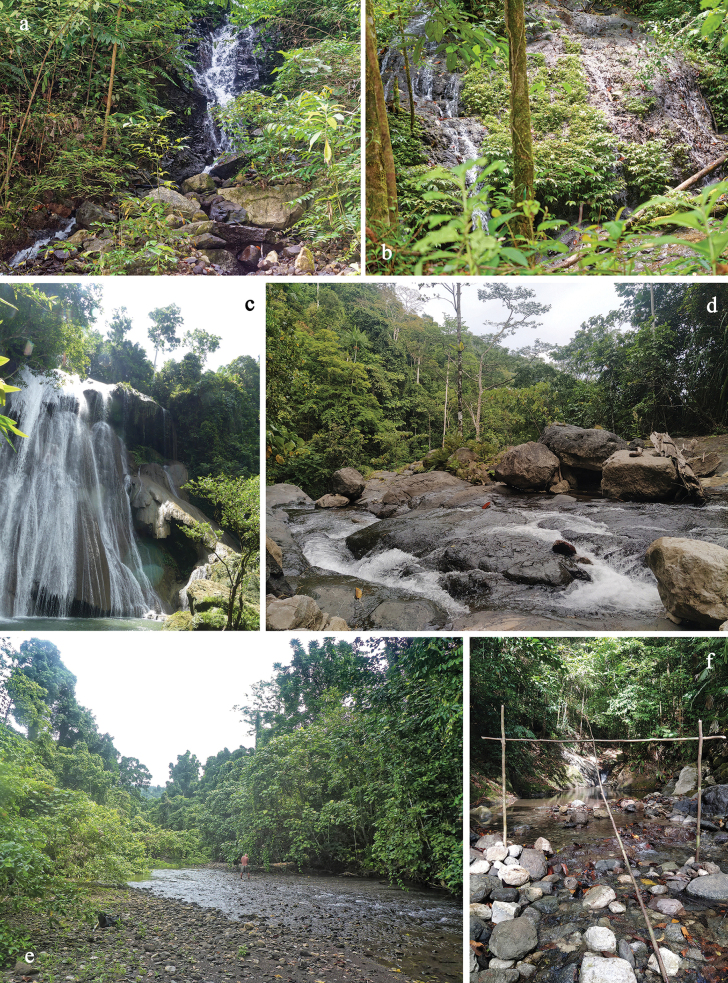
Habitats: *P. batantaraja* sp. nov. (a. Type locality, b); *P. cukiclara* sp. nov. (a, b, d. Type locality); *P. cataracta* sp. nov. (c. type locality); *P. batanlenos* sp. nov. (a, d. Type locality, e, f); *P. horvathrobi* sp. nov. (e); *P. longabranchias* sp. nov. (d. Type locality, f).

The species is known from two watercourses in southern Batanta: Kalijakut (Fig. 29d (type locality), f) and Tanjung Lampu Rivers at elevations from 182–420 m. It co-occurs with larvae of *P. batanlenos* sp. nov. and *P. cukiclara* sp. nov.

#### Etymology.

The species name *longabranchias*, meaning “long gills” in Latin, refers to the very long and slender shape of the tergalii.

#### Distribution.

**Figure 30.**
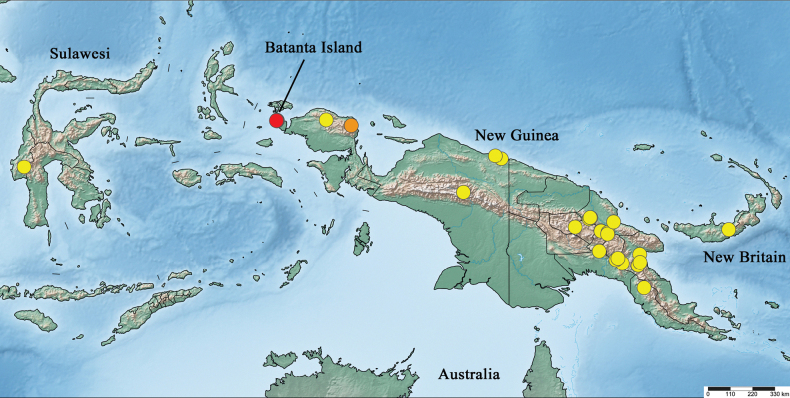
Distribution of *Papuanatula* species: yellow dots: known species (Kaltenbach et al. 2025); orange dot: *Papuanatula arfak* sp. nov. (present study); red dots: species from Batanta – *P. batantaraja* sp. nov., *P. cukiclara* sp. nov., *P. cataracta* sp. nov., *P. batanlenos* sp. nov., *P. horvathrobi* sp. nov., *P. longabranchias* sp. nov. (present study).

New Guinea, Batanta Island (Fig. 30).

##### ﻿Key to the species of subgenus Papuafiliola from New Guinea (larvae)

Based on Kaltenbach et al. 2025.

**Table:** 

1	Abdominal terga without protuberances	**2**
–	Abdominal terga with median protuberances	***P. tuberculata***
2(1)	Femur with brown, hypodermal marking in large blank on anterior side; tergalii very long and narrow	**3**
–	Femur without marking in large blank on anterior side; tergalii oval	***P. horvathrobi* sp. nov.**
3(2)	Marking on femur shoe-shaped, usually longer than wide; surface of abdominal terga without fine, simple setae	***P. longabranchias* sp. nov.**
–	Marking on femur usually wider than long; surface of abdominal terga with fine, simple setae	***P. stenophylla***

## ﻿Discussion

### ﻿Diversity of *Papuanatula*

Previously, 26 species of Papuanatula were reported, 24 of the subgenus Papuanatula s. str. and two of the subgenus Papuafiliola (Fig. 30; Lugo-Ortiz and McCafferty 1999; Kaltenbach et al. 2025). Here, we describe one additional species from the main island of New Guinea and six new species from the rather small island of Batanta, five of the subgenus Papuanatula s. str. and two of the subgenus Papuafiliola. The species from Batanta were found during repeated, intensive collections in the past few years. It should be noted that many water courses were not surveyed yet, and no collections were made above an elevation of 550 m. The distribution of the six new species across the sampled water systems is shown in Table 2. The Kalijakut River system, which originates at the highest point in Batanta, has the largest number of *Papuanatula* species, four. It also has the highest diversity of Trichoptera species (Kovács et al. 2025).

**Table 2.** Distribution of *Papuanatula* species in different river systems of Batanta.

	Batanta north side				Batanta south side		
	Waridor	Warai	Warinkabon	Waibin	Tanjung Lampu	Kalijakut	Wailebet
*P. batantaraja* sp. nov.						x	
*P. cukiclara* sp. nov.						x	
*P. cataracta* sp. nov.			x				
*P. batanlenos* sp. nov.	x				x	x	x
*P. horvathrobi* sp. nov.	x	x		x			
*P. longabranchias* sp. nov.					x	x	

Areas or river systems studied in more detail in New Guinea have often revealed a composition of several co-occurring species, different from other areas or river systems. Examples are: the Je River (Arfak Mountains) with *P. arfak* sp. nov., *P. dumspinae*, *P. paratuber*, *P. epituber*, and *P. pilosa*; the Bulolo River near Wau with *P. bessa* Lugo-Ortiz & McCafferty, *P. tuber* Lugo-Ortiz & McCafferty, and *P. plana* Lugo-Ortiz & McCafferty; the Elagaima River (Baliem Valley) with *P. obscura* Kaltenbach, Kluge & Gattolliat, *P. obscurella* Kaltenbach, Kluge & Gattolliat, *P. tuberculata* Kaltenbach, Kluge & Gattolliat, and *P. zebrata* Kaltenbach, Kluge & Gattolliat; the Cyclops Mountains with *P. bessa*, *P. cyclopomontana* Kaltenbach, Kluge & Gattolliat, *P. heterochaeta* Kaltenbach, Kluge & Gattolliat, and *P. stenophylla* (Kaltenbach et al. 2025). This gives an idea of the enormous diversity of *Papuanatula* in New Guinea, and the number of additional species we may discover with further collection activities in New Guinea and the surrounding archipelago.

### ﻿Genetics

COI barcode sequences were obtained from five different species, four from the subgenus Papuanatula s. str. and one from the subgenus Papuafiliola (Table 1). The interspecific distance is always between 16% and 25% (K2P), which confirms the values reported by Kaltenbach et al. 2025 for *Papuanatula* in New Guinea (19%–25%, K2P). The intraspecific distances are always between 0% and 1%, as expected. See also discussion in Kaltenbach et al. 2025.

## Supplementary Material

XML Treatment for Papuanatula

XML Treatment for Papuanatula (Papuanatula) arfak

XML Treatment for Papuanatula (Papuanatula) batantaraja

XML Treatment for Papuanatula (Papuanatula) cukiclara

XML Treatment for Papuanatula (Papuanatula) cataracta

XML Treatment for Papuanatula (Papuanatula) batanlenos

XML Treatment for Papuafiliola

XML Treatment for Papuanatula (Papuafiliola) horvathrobi

XML Treatment for Papuanatula (Papuafiliola) longabranchias

## ﻿Additional information

### Use of AI

No use of AI was reported.

### Author ORCIDs

Thomas Kaltenbach https://orcid.org/0000-0001-8052-0388

Tibor Kovács https://orcid.org/0000-0003-4577-0394

Jean-Luc Gattolliat https://orcid.org/0000-0001-5873-5083

### Data availability

All of the data that support the findings of this study are available in the main text.
